# Microbial Communities and Physicochemical Properties of the Nile River Water in the Suez Canal Area

**DOI:** 10.3390/microorganisms13102395

**Published:** 2025-10-19

**Authors:** Noha Elkayal, Samira Zakeer, Marwa Azab, Ali Abdellah, Sarah Shabayek

**Affiliations:** Department of Microbiology and Immunology, Faculty of Pharmacy, Suez Canal University, Ismailia 41522, Egypt; noha.elkayal@gmail.com (N.E.);

**Keywords:** freshwater, microbiome, River Nile, Ismailia Canal, 16S RNA, functional prediction

## Abstract

Monitoring freshwater resources is crucial to drinking water quality. The Ismailia Canal supplies most freshwater to the Suez Canal area in Egypt. However, information on the freshwater microbiome is limited in this region. A total of 59 freshwater samples were collected. Along with determining the physicochemical properties of the samples, we used conventional methods to identify indicator bacteria. To overcome limitations of conventional culture, we employed high-throughput 16S rRNA gene sequencing, taxonomy profiling, and functional prediction to study uncultivated microbial communities. Total and fecal coliforms prevailed in 100% and 80% of samples, respectively. Predominant contaminants included *E. coli*, fecal streptococci, *Pseudomonas aeruginosa*, and *Staphylococcus aureus*. Taxonomic profiling revealed dominance of *Proteobacteria* and *Actinobacteriota*. *Proteobacteria* showed a positive correlation with *Bacteroidetes* and a negative correlation with *Actinobacteria*. Most samples had similar bacterial community structures, despite location-driven variability. Elevated bacterial loads were notable at the Qassasin district, which exhibited the highest relative abundance of genes associated with bacterial infections. This study provides key insights into the impact of freshwater microbiome on public health.

## 1. Introduction

Clean drinking water is always necessary for human health, and an adequate supply must be available to consumers. Diseases related to water claim more than 829,000 lives annually on a global scale [1]. According to the WHO, comprehensive approaches to risk assessment and management of a drinking water supply enhance confidence in the safety of water [2]. The quality of water and the purpose of use can be determined and evaluated by certain physical, biological, and chemical parameters [3].

The Nile River is the main source of life in Egypt, accounting for more than 90 percent of the freshwater supply [4]. The Ismailia Canal, one of the main River Nile branches, is the primary source of freshwater for several governorates, cities, and villages in the East of Egypt. It branches from the Nile at Cairo and runs straight to the East toward the Timsah Lake in Ismailia, passing by the Qalioubeya and Sharqeya governorates [5]. At Ismailia, the Ismailia Canal bifurcates into two arms (Figure S1): one to the North to supply Port-Said passing by Qantara-Gharb district (Port-Said Canal), and the second to the South to supply Suez passing by Fayed district (Suez Canal) [6,7]. In addition, there is a small branch of the Ismailia Canal called Manayef Canal, which runs parallel to it to the Manayef district (Figure S2). Finally, there is a linkage canal connecting both the Ismailia Canal and Suez Canal, known as the Sinai Canal (Figure S2). Moreover, a large wastewater treatment plant, the Al-Mahsama station (Figure S3), was constructed in 2020 in the Sinai Peninsula’s Eastern Suez Canal region [8] to collect, treat, and transfer agricultural drainage water through the Serapeum siphon under the New Suez Canal to Al-Abtal Canal on the Eastern side of the Suez Canal [9]. Al-Abtal Canal ends in the Huda Canal in Qantara Sharq, for agricultural use in Sinai [10].

Ismailia Canal, along with its branches, is exposed to a considerable deterioration in water quality because of different wastes that are discharged into the water stream [11]. The poorly structured drainage and sewage systems and the considerable number of industries may lead to increased discharges of fecal microorganisms and heavy metals through wastewater into fresh waterways [12]. Indicator bacteria like total coliforms, fecal coliforms, and fecal streptococci are frequently used to assess fecal pollution and the potential deterioration of water quality in freshwater sources [13]. *Escherichia coli*, *Enterococcus faecalis*, *Pseudomonas aeruginosa*, and *Staphylococcus aureus* are among the most common bacteria found in the surface water of the River Nile [14,15].

Metagenomics allows the study of microbial communities taken directly from their natural environment without prior cultivation, providing a way to characterize uncultured microbes in their natural habitat [16]. Metagenomic methods can be used to characterize the water microbiome and consequently determine the potential sources of aquatic microbial contamination [17]. This may help determine the quality of surface water and lead to more effective drinking water treatment in the future [18].

The continuous assessment of freshwater resources is imperative due to their direct influence on drinking water quality. Previous research predominantly employed traditional cultural methods to assess the bacterial quality of the Nile water [13,14,15,19,20,21,22,23,24]. With the exception of the study by Eraqi et al. 2018 [25] which examined the water microbiome of the Nile in Cairo, investigations into the microbiome of the Nile water remain limited. To our knowledge, this study is the first to characterize the microbiome of freshwater resources in the Suez Canal region. We examined the physicochemical and bacteriological characteristics of freshwater canals in the Ismailia governorate. Furthermore, the freshwater microbiome was characterized using 16S rRNA next-generation sequencing technology. This approach enabled us to identify potential roles of dominant bacteria and to understand how microbial community composition and functional profiles may influence the aquatic ecosystem’s response to environmental changes or anthropogenic effects.

## 2. Materials and Methods

### 2.1. Study Area and Sample Collection

**Figure 1:**
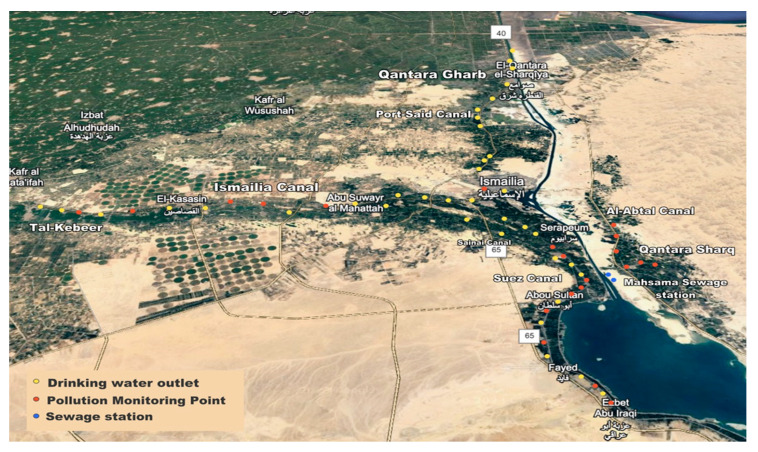
A map illustrating the sampling sites of raw surface water samples. Yellow circles indicate drinking water intakes, red circles indicate pollution monitoring points, and blue circles indicate Al-Mahsama sewage station.

Sample collection was performed from May to June 2021. Collection points are anticipated in Figure 1, and a detailed summary of collected samples, location coordinates, and sampling time points is shown in Table S1.

A total of 59 raw surface water samples, covering freshwater canals in all districts of the Ismailia governorate, were collected as follows. Ismailia contains a total of 37 freshwater treatment plants, which receive raw water from drinking water intakes that cover the seven districts of Ismailia governorate (Ismailia, Fayed, Abo-soir, Qasassin, Tal-Kebeer, Qantara Gharb, Qantara Sharq) and are located on 6 freshwater canals (Ismailia, Suez, Port-Said, Al-Abtal, Manayef, Sinai). A total of 37 samples were collected from the drinking water intakes of drinking water treatment plants. In addition, 20 more samples were collected from 20 pollution monitoring sites covering the 6 freshwater canals. These points are designated by the Egyptian water treatment central laboratories based on industrial, agricultural, human activity, and pollution resources. Finally, 2 samples were collected from the inlet and outlet of the Al-Mahsama wastewater treatment station. Raw surface water samples (3 L) were collected using sterile glass bottles by positioning the container’s mouth against the direction of the water flow, leaving a gap in the upper third of the sample bottle. Samples were transported on ice and analyzed within 3 h [26]. Raw surface water samples were divided into 2 unequal portions; one portion (2 L) was used for physicochemical analysis, and the other portion (1 L) was used for bacteriological and microbiome analysis.

### 2.2. Physicochemical Characters

The following physicochemical parameters were determined for each raw water sample: temperature, pH, total dissolved solids (TDS), biochemical oxygen demand (BOD), dissolved oxygen (DO), ammonia (NH_3_), nitrate (NO_3_), nitrite (NO_2_), chloride (Cl), calcium hardness (CaH), magnesium hardness (MgH), calcium (Ca), magnesium (Mg), total hardness (TH), and total alkalinity (T.Alk). For sewage samples, only temperature, BOD, and DO were measured as recommended by the Egyptian Law 48/1982 for the Protection of the River Nile and Waterways from Pollution. All measurements were taken as per Standard Methods described for the Examination of Water and Wastewater by the American Public Health Association, the American Water Works Association, and the Water Environment Federation [26]. The reference range for parameters was according to the Egyptian Law 48/1982 for the Protection of the River Nile and Waterways from Pollution [27], the Egyptian Standards for Potable Water as outlined in Egyptian Law 458/2007 [28], and the WHO guidelines for drinking water quality [29].

### 2.3. Bacteriological Analysis

Raw water samples were examined for the presence of classical bacterial indicators for pollution; total coliforms, fecal coliforms, and fecal streptococci, according to Standard Methods for the Examination of Water and Wastewater [26]. The fecal coliform test was not applied to sewage samples as per the guidelines of the Egyptian Law 48/1982 for the Protection of the River Nile and Waterways from Pollution [27]. In addition, raw water samples were screened for the presumptive detection and isolation of *P. aeruginosa*, *S. aureus*, *E. coli*, and fecal streptococci. For *P. aeruginosa*, samples were inoculated in Tryptic Soy broth, incubated for 24 h at 35 ± 2 °C, then subcultured on Cetrimide agar (HiMedia Laboratories, Maharashtra, India). Colonies showing green pigmentation confirmed the presence and isolation of *P. aeruginosa* [30]. For *S. aureus*, samples were subcultured on Mannitol Salt agar (HiMedia Laboratories, Maharashtra, India). Yellow colonies with yellow zones confirmed the presence and isolation of *S. aureus* [31]. For *E. coli*, positive broth tubes from the fecal coliform test were subcultured and examined for pink colonies on MacConkey agar (HiMedia Laboratories, Maharashtra, India). These were further examined on Eosin Methylene Blue agar (HiMedia Laboratories, Maharashtra, India). Colonies with a metallic green sheen confirmed the presence and isolation of *E. coli* [32]. For fecal streptococci, positive Azide-Dextrose broth tubes from the fecal streptococci test were subcultured on Bile Esculin Azide agar (HiMedia Laboratories, Maharashtra, India). Brownish-black colonies with brown halos confirmed the presence and isolation of fecal streptococci [26]. All isolates were subjected to further identification using the Automated ID and AST System MA120 (Render Biotech Co., Shenzhen, China). The MA120 System functions through integrated colorimetric and turbidimetric methodologies to achieve reliable microbial identification by utilizing biochemical reaction-based color changes.

### 2.4. Microbiome Analysis

#### 2.4.1. Sample Preparation

Raw surface water samples (500 mL), used for bacteriological analysis, were filtered through a sterile 0.2 µm cellulose nitrate membrane and then transferred into sterile collection tubes containing 5 mL of phosphate-buffered saline (Sigma, Darmstadt, Germany). Membrane filters were stored at −20 °C until DNA extraction [18].

#### 2.4.2. DNA Extraction

Collection tubes were vortexed at their maximum speed for 10 min. DNA was extracted using the DNeasy^®^ PowerSoil^®^ Kit (QIAGEN, Germantown, MD, USA), following manufacturer instructions. A NanoDrop Spectrophotometer (Thermofisher Scientific, Waltham, MA, USA) was used to calculate the concentration of DNA. The integrity of the DNA was then assessed using agarose gel electrophoresis. DNA was stored at −20 °C until being processed.

#### 2.4.3. PCR Amplification of 16S rRNA Gene and Illumina MiSeq Sequencing

PCR was performed using primer sequences targeting the 16S rRNA V3–V4 region, as described by [33]. PCR was performed in a BIORAD T100 thermal cycler using the following program: 95 °C for 3 min, followed by 25 cycles of: 95 °C for 30 s, 55 °C for 30 s, 72 °C for 30 s, then final extension at 72 °C for 5 min. After amplification, gel electrophoresis with a 100 bp ladder was performed on a 2% agarose gel to verify the size and quality of PCR products (Figure S4). PCR amplicons were sent for library preparation and paired-end 2 × 300 MiSeq illumina sequencing at IGA Technology Services, Udine, Italy. Negative controls without DNA templates were included to detect the presence of contaminants in the extraction reagents, and during the PCR mixture setup up and to reduce the likelihood of contamination.

#### 2.4.4. Manipulation of Raw Sequence Data

FASTQC (0.11.9) and MultiQC (version 1.13) packages were used for quality inspection and to determine the appropriate trimming positions to be used in downstream steps [34]. Raw data analysis was performed using Quantitative Insights into Microbial Ecology 2 (QIIME 2 version 2022.8) [35]. Raw paired-end demultiplexed sequences were imported into QIIME 2. The Divisive Amplicon Denoising Algorithm 2 (DADA2) plugin was used for quality filtering, trimming, denoising, chimera removal, and merging paired reads [36]. Based on the quality score profiles, forward and reverse reads were truncated at 280 bp and 220 bp, respectively, to keep a Q30 median across samples and remove low-quality base calls near the read ends while preserving sufficient overlap for merging. All other DADA2 parameters were set to defaults: no trimming from the 5′ end; maximum expected errors (max-ee) = 2.0 for both forward and reverse reads; truncation quality score (trunc-q) = 2; minimum overlap = 12 bp; chimera removal method = consensus; and pooled sample inference disabled. The output of the DADA2 plugin includes the ASV (Amplicon Sequence Variants) table with the representative sequences. A phylogenetic tree was built by carrying out multiple sequence alignments using the “Mafft” plugin in QIIME 2 [37], then masking (or filtering) the alignment to remove highly variable positions. The unrooted tree was created using the “Fasttree” plugin in QIIME 2 and rooted using the longest root. Rarefaction curves were then generated to assess the sampling depth [38].

#### 2.4.5. Alpha and Beta Diversity

The rooted phylogenetic tree served as the foundation for calculating basic metrics used in the analysis of alpha and beta diversity. To minimize the bias from varying sequencing depth, phyloseq rarefaction was performed before calculating the diversity metrics [39,40]. Rarefaction curves were generated using the Qiime diversity alpha-rarefaction plugin; the maximum sample depth used was 5500. Alpha diversity describes taxa diversity within samples. Alpha diversity metrics assessed included Shannon, observed features, Chao1, Simpson, ACE, and Fisher indices. The significance of these alpha diversity indices was determined using the Kruskal–Wallis test with the “kruskal.test” method and the “stat_compare_means” R function [41]. For comparisons between individual sample groups, pairwise Wilcoxon rank-sum tests were performed with the “pairwise.wilcox.test” R function, and results were adjusted using the Benjamini–Hochberg (BH) procedure [42]. Statistical significance was defined as *p*-values ≤ 0.05. The alpha diversity indices and significance tests were conducted using the “Phyloseq (version 1.42.0), dplyr (version 2.3.3), microbiome (version 1.20.0), tidyverse (version 2.0.0), DESeq2 (version 1.38.3), vegan (version 2.6-4), and stats (version 4.2.2)” R packages, while plots were generated using the “ggpubr (version 0.6.0)” and “ggplot2 (version 3.4.3)” R packages. Beta diversity describes variation in community composition between samples. Principal Coordinate Analysis (PCoA) plots were generated from: Bray–Curtis distance, Jaccard distance, weighted UniFrac distance, and unweighted UniFrac distance. The statistical significance between groups was assessed using a permutation-based ANOVA (PERMANOVA) test–ADONIS and 999 permutation-based distance metrics [42]. To determine whether within-group distances or dissimilarities are greater than or equal to between-group distances using the ranks of all pairwise sample distances, PERMANOVA pairwise contrast was performed using the R function “pairwise.adonis” [41]. “Phyloseq (version 1.42.0), dplyr (version 2.3.3), microbiome (version 1.20.0), tidyverse (version 2.0.0), DESeq2 (1.38.3), vegan (version 2.6-4), and stats (version 4.2.2) R” packages were used to estimate the beta diversity metrics and PERMANOVA tests, while the “ggplot2 (version 3.4.3)” package was used for generating the plots.

#### 2.4.6. Taxonomy Assignment and Core Microbiome

The ASV feature table and phylogenetic tree files were exported from QIIME 2, and taxonomy assignment of ASV sequences was performed using the Bayesian Least Common Ancestor (BLCA) approach, which is a software implemented as a Python package using the SILVA138 database. The following packages were installed for the BLCA approach: “biopython (version 1.79), BLAST (version 2.9.0), CLUSTALO (version 1.2.6), and MUSCLE (version 5.1)” [43,44]. The QIIME 2 output files (feature table, phylogenetic tree, and taxonomy file) were then uploaded to the “MicrobiomeAnalyst” platform [45].

#### 2.4.7. Linear Discriminant Analysis Effect Size (LEfSE)

Linear discriminant analysis effect size (LEfSE) was used to identify the generated taxa that differed among the various sampling sites and to ascertain whether any of these genera were potentially pathogens or signs of potential fecal contamination [17]. This is a three-step process: first, a non-parametric Kruskal-Wallis is used to find taxa that were significantly different between sources; next, pairwise Wilcoxon rank-sum tests are used on these taxa; and finally, linear discriminant analysis (LDA) is used to figure out the effect size and biological consistency within the groups being tested. LDA score ≥ 2 and a *p*-value ≤ 0.05 were considered statistically significant. The “MicrobiomeAnalyst” platform [45] was used to perform this step.

#### 2.4.8. Correlation Analysis

In order to interpret the structure of complex microbial communities, network analysis of significant taxa co-occurrence using Spearman correlation patterns was used [46]. We performed the correlation network using the “MicrobiomeAnalyst” platform [45] on different taxonomic levels: phylum, family, genus, and species.

#### 2.4.9. Pathway and Functional Prediction

The “Tax4fun2” R-based tool (version 1.1.5) was used for the prediction of functional profiles and functional redundancy of the bacterial communities [47]. A BLAST search against the SILVA database (SILVA_123_SSURef_Nr99) was used to taxonomically classify ASV sequences. Functional profiles were then calculated based on the obtained protein sequences with UProC version 1.2.0 [48] using the KEGG Orthology (KO) database for prokaryotes (July 2018 release of the Kyoto encyclopedia of genes and genomes) [49] as a reference. The level 1, 2, and 3 pathway predictions were detected using the “Tax4Fun2” package, and significant differences between sample groups were calculated using the Kruskal–Wallis rank sum test through the “stat_compare_means” R function. The highest level 1 and 2 functions underwent additional analysis in order to identify the most crucial factors that may have an impact on the quality of the raw water samples. Python packages “Pandas (version 1.5.0), statistics (version 1.20.0), numpy (version 1.20.0), and pylab (module from Matplotlib version 3.5.0)” were used to perform statistical analysis for pathway and functional prediction tables, while “Matplot (version 3.5.0), Seaborn (version 0.11), and Plotly (version 5.0.0)” were used to generate plots beside the “ggpubr (version 0.6.0) and ggplot2 (version 3.4.3)” R packages. As a potentially significant indicator of how resilient a community will be to environmental changes, the functional redundancy index (FRI) was calculated [47]. FRI is one of the “Tax4Fun2” tool outputs. Python packages “Pandas (1.5.0), Statistics (version 1.20.0), Numpy (version 1.20.0), and PyLab (module from Matplotlib version 3.5.0)” were used for statistical analysis of FRI to divide values into low, moderate, and high values. Plots were generated using the Python packages “matplot (version 3.5.0)”, “seaborn (version 0.11)”, and “plotly (version 5.0.0)” and the R packages “ggpubr (version 0.6.0)” and “ggplot2 (version 3.4.3)”.

#### 2.4.10. Data Availability

Raw reads were deposited in the NCBI database with SRA under Bio project Accession number PRJNA1074353, which is accessible at http://www.ncbi.nlm.nih.gov/bioproject/1074353 (accessed on 7 February 2024).

## 3. Results

### 3.1. The Physicochemical Parameters

The observed physicochemical parameters of raw surface water samples were as follows. The temperature ranged from 24.3 to 28.2 °C with an average of 25.7 ± 0.7 °C. According to the WHO [29], high water temperatures can exacerbate issues related to taste, odor, color, and corrosion, as well as promote microbial growth, although specific thresholds for water temperature are not provided. The pH ranged from 6.7 to 8.6 with an average of 7.7 ± 0.5, which is compatible with Egyptian drinking water quality standards.

The TDS ranged from 4.4 to 238 mg/mL with an average of 225.1 ± 42 mg/L. The T.ALK ranged from 88 to 188 mg/L CaCO_3_ with an average of 160.6 ± 15.3 mg/L CaCO_3_. Both TDS and T.ALK values were within the permissible limits.

**Figure 2:**
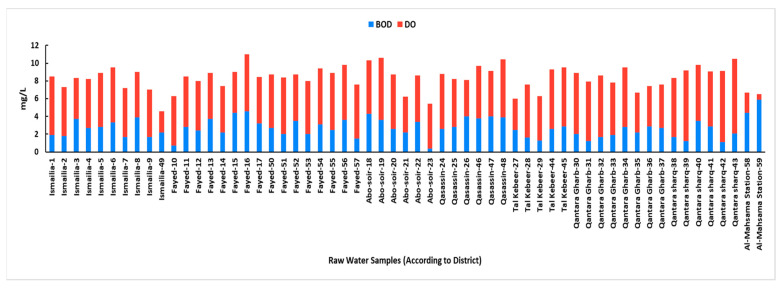
Levels of Biochemical oxygen demand (BOD) and dissolved oxygen (DO) in raw surface water samples according to sampling district. All measurements were taken as per Standard Methods described for the Examination of Water and Wastewater by the American Public Health Association, the American Water Works Association, and the Water Environment Federation.

The DO ranged from 0.6 to 8.4 mg/L with an average of 5.6 ± 1.3 mg/L. All samples were within the permissible limits (≥5 mg/L) except for 8 samples from the drinking water intakes (Sample 3 from Ismailia, Sample 15 from Fayed, Sample 21 from Abo-Soir, Sample 26 from Qasassin, Sample 27 from Tal kabeer, Samples 35, 36, and 37 from Qantara Gharb, DO values from 3.5 to 4.9 mg/L), one sample from the pollution monitoring points (Sample 49 from Ismailia, DO value of 2.4 mg/L) and the 2 samples of the Al-Mahsama wastewater treatment station (Samples 58 and 59, DO values of 2.3 and 0.6 mg/L, respectively). BOD ranged from 0.4 to 5.9 with an average of 2.7 ± 1.1 mg/L, which is within the permissible limits. However, the BOD was notably elevated in samples from the Qasassin and Fayed districts and along with Al-Mahsama station points, with DO values generally inversely related to BOD across most samples (Figure 2).

**Figure 3:**
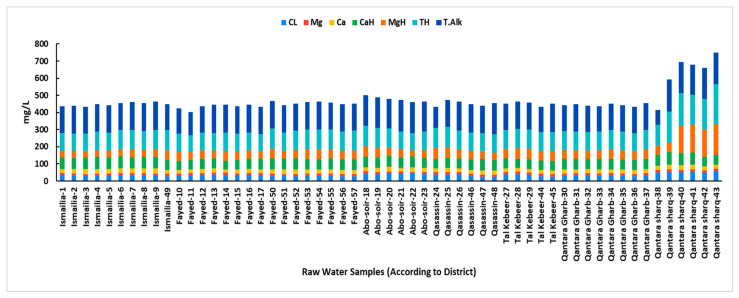
Levels of Chloride (CL), Magnesium (Mg), Calcium (Ca), Calcium hardness (CaH), Magnesium hardness (MgH), Total hardness (TH), and Total alkalinity (T.Alk) in raw surface water samples according to sampling district. For sewage samples, only temperature, BOD, and DO were measured as recommended by the Egyptian Law 48/1982 for the Protection of the River Nile and Waterways from Pollution. All measurements were taken as per Standard Methods described for the Examination of Water and Wastewater by the American Public Health Association, the American Water Works Association, and the Water Environment Federation.

The TH ranged from 100 to 232 mg/L with an average of 117.6 ± 24.5 mg/L. The magnesium levels ranged from 8.16 to 14.9 mg/L with an average of 11.9 ± 1.8 mg/L, whereas calcium levels ranged from 19.2 to 28.8 mg/L with an average of 24.6 ± 2 mg/L. The CaH ranged from 48 to 72 mg/L with an average of 61.4 ± 5.2 mg/L. The MgH ranged from 34 to 178 mg/L with an average of 57.6 ± 30.6 mg/L. The chloride levels ranged from 26 to 57 mg/L with an average of 35.9 ± 7.4 mg/L. Except for MgH, the values of TH, magnesium, calcium, CaH, and chloride were all within permissible limits. However, the magnesium concentrations were significantly high at all pollution monitoring points in the Qantara Sharq district, sourced from the Al-Abtal Canal. The same scenario was observed with MgH values (Samples 40, 41, 42, and 43, MgH ranged from 160 to 178 mg/L), which were all above the permissible limits (≤150 mg/L). These samples were also obtained from the pollution monitoring points at the Qantara Sharq district, sourced from the Al-Abtal Canal. This was coupled with elevated chloride levels, likely a result of the region’s low water levels, contributing to increased overall water hardness and magnesium hardness (Figure 3).

**Figure 4:**
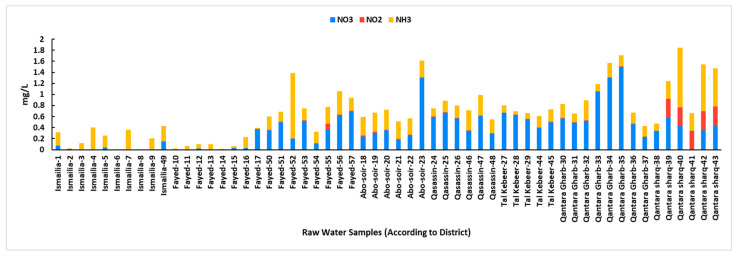
Levels of Nitrate (NO^3−^), Nitrite (NO^2−^), and Ammonia (NH_3_) in raw surface water samples according to sampling district. For sewage samples, only temperature, BOD, and DO were measured as recommended by the Egyptian Law 48/1982 for the Protection of the River Nile and Waterways from Pollution. All measurements were taken as per Standard Methods described for the Examination of Water and Wastewater by the American Public Health Association, the American Water Works Association, and the Water Environment Federation.

The NO_3_ ranged from 0.02 to 1.5 mg/L with an average of 0.5 ± 0.3 mg/L, which is within the permissible limits. Generally, the NO_3_ concentrations were particularly high in samples from drinking water intakes in the Port-Said Canal within the Qantara-Gharb district, followed by samples from drinking water intakes in the Ismailia Canal within the Qasassin and Tal kabeer districts, while minimal NO_3_ presence could be observed in several samples from Ismailia, Fayed, and Qantara Sharq. The NO_2_ levels ranged from 0.001 to 0.4 mg/L with an average of 0.04 ± 0.1 mg/L. All samples were within the permissible limits (≤0.2 mg/L) except for 5 samples (Samples 39, 40, 41, 42, and 43, NO_2_ levels from 0.331 to 0.354 mg/L) from the pollution monitoring points at the Qantara-Sharq district were alarmingly elevated. Again, all these five samples were sourced from the Al-Abtal Canal. The NH_3_ levels ranged from 0.01 to 1.18 mg/L with an average of 0.3 ± 0.2 mg/L. All samples were within the permissible limits (≤0.5 mg/L) except for 4 samples (Samples 40, 42, 43, and 52, NH_3_ levels from 0.69 to 1.18 mg/L) from the pollution monitoring points, three of them were at the Qantara Sharq district sourced from the Al-Abtal Canal, and one at the Fayed district sourced from the Suez Canal. Notably, the NH_3_ and NO_2_ concentrations were relatively low in samples with elevated NO_3_ levels. The NO_3_, NO_2_, and NH_3_ levels in raw surface water samples are shown in Figure 4. Detailed readings of all physicochemical parameters of raw surface water samples are shown in Table S2.

### 3.2. Total and Fecal Coliform Enumeration and Potential Detection of Common Bacterial Contaminants

Total coliforms were detected in all raw water samples; values ranged from 20 to 16,000 MPN/100 mL. All samples were within permissible limits (1000 MPN/100 mL) except for eight samples, including the two samples from the Al-Mahsama wastewater treatment station, where the highest loads were observed (Samples 58 and 59 with values of 2400 and 16,000 MPN/100 mL, respectively). The remaining six samples included four samples from the drinking water intakes (Sample 8 at Ismailia district from Port-Said Canal, Sample 16 at the Fayed district from Suez Canal, Samples 25 and 26 at the Qassasin district from Ismailia Canal,) and two samples from the pollution monitoring points (Sample 48 at the Qassasin district from the Ismailia Canal and Sample 52 at the Fayed district from the Suez Canal).

**Figure 5:**
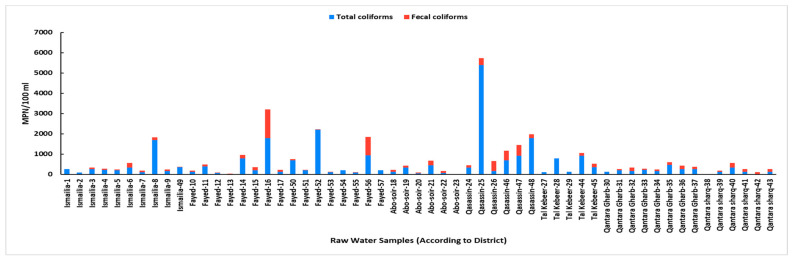
Total and fecal coliform counts of raw surface water samples according to sampling district. Total coliforms were detected in all raw water samples. The fecal coliform test was not applied to sewage samples as per the guidelines of the Egyptian Law 48/1982 for the Protection of the River Nile and Waterways from Pollution.

Fecal coliforms were detected in 80% (47 out of 59) of raw water samples. Values ranged from 20 to 1400 MPN/100 mL. All samples were within permissible limits (200 MPN/100 mL) except for eight samples, including four samples (Samples 25, 26, 46, and 47) at the Qassasin district from Ismailia Canal (two from the drinking water intakes and two from the pollution monitoring points). The remaining four samples included three samples (Samples 6, 16, and 21) from drinking water intakes at Ismailia (sourced from Sinai Canal), Fayed (sourced from Suez Canal), and Abo-Soir (sourced from Manayef Canal) districts, and one sample (Sample 40) from the pollution monitoring point at Qantara Sharq district (sourced from Al-Abtal Canal). The presence of fecal coliforms was directly proportional to the total coliform counts (Figure 5). Of note, Spearmen correlation revealed that BOD positively correlates with total and fecal coliforms, while chloride negatively correlates with total and fecal coliforms.

The presumptive detection and isolation of common bacterial contaminants; *P. aeruginosa*, *S. aureus*, *E. coli*, and fecal streptococci, was also performed (Figure S5). For *P. aeruginosa*, 29 out of 59 (49.2%) water samples gave green pigmentation on Cetrimide agar (5/10 samples from Ismailia, 9/16 samples from Fayed, 2/6 samples from Abo-Soir, 6/6 samples from Qasassin, 2/5 samples from Tal kabeer, 4/8 samples from Qantara Gharb, and 1/6 sample from Qantara Sharq). For *S. aureus*, 45 out of 59 (76.3%) water samples produced yellow colonies with yellow zones on Mannitol Salt agar (8/10 samples from Ismailia, 11/16 from Fayed, 4/6 samples from Abo-Soir, 6/6 samples from Qasassin, 2/5 samples from Tal kabeer, 7/8 samples from Qantara Gharb, 5/6 from Qantara Sharq, and the 2 samples from the Al-Mahsama wastewater treatment plant). For *E. coli*, 30 out of 59 (50.8%) water samples gave green metallic sheen on Eosin Methylene Blue agar (5/10 samples from Ismailia, 9/16 from Fayed, 2/6 from Abo-Soir, 6/6 samples from Qasassin, 5/8 samples from Qantara Gharb, 2/6 samples from Qantara Sharq, and 1/2 sample (inlet) from the Al-Mahsama wastewater treatment plant). For fecal streptococci, 47 out of 59 (79.7%) water samples gave brownish-black colonies with brown halos on Bile Esculin Azide agar (6/10 samples from Ismailia, 11/16 samples from Fayed, 6/6 samples from Abo-Soir, 6/6 samples from Qasassin, 2/5 samples from Tal kabeer, 8/8 samples from Qantara Gharb, 6/6 samples from Qantara Sharq, and the 2 samples from the Al-Mahsama wastewater treatment plant). Notably, all six samples obtained from the Qasassin district were positive for *P. aeruginosa*, *S. aureus*, *E. coli*, and fecal streptococci.

### 3.3. Sequence Analysis

Illumina sequencing results produced 7,481,990 paired-end reads from 47 samples (5 out of 59 original samples failed the sequencing process, and 7 samples were removed from the analysis process due to the low number of reads). After quality filtering, 2,917,889 reads remained, with a minimum of 22,922 and a maximum of 220,252 reads per sample. After merging reads and chimera removal, 420,950 reads were produced, and the library size ranged from 2506 to 19,461 reads, with an average of 8957 reads per sample. Approximately 6198 ASVs with an average read length of 453 bp were used for downstream taxonomic analyses.

#### 3.3.1. Alpha Diversity

**Figure 6:**
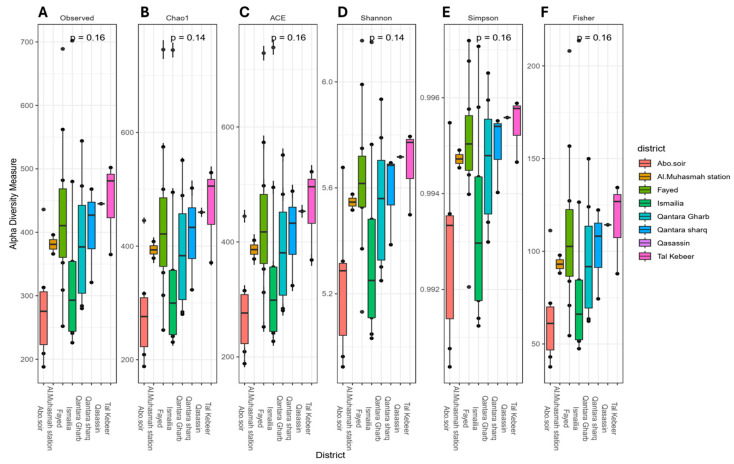
A boxplot illustrating alpha diversity of raw surface water samples with respect to sampling district as revealed by the alpha diversity indices: (**A**) Observed features, (**B**) Chao1, (**C**) ACE, (**D**) Shannon, (**E**) Simpson, and (**F**) Fisher. The line inside each box represents the median value. Outliers are shown as dots. BH-adjusted *p*-values were used to indicate significance. The Kruskal–Wallis test was used to assess the significance at *p*-value ≤ 0.05.

Rarefaction curves for samples were generated using the vegan R package (Figure S6). The maximum sample depth used was 5500. There was no significant difference in alpha diversity with respect to the sample district (Figure 6). Additionally, no significant results were found in Wilcoxon rank-sum pairwise comparison tests.

#### 3.3.2. Beta Diversity

**Figure 7:**
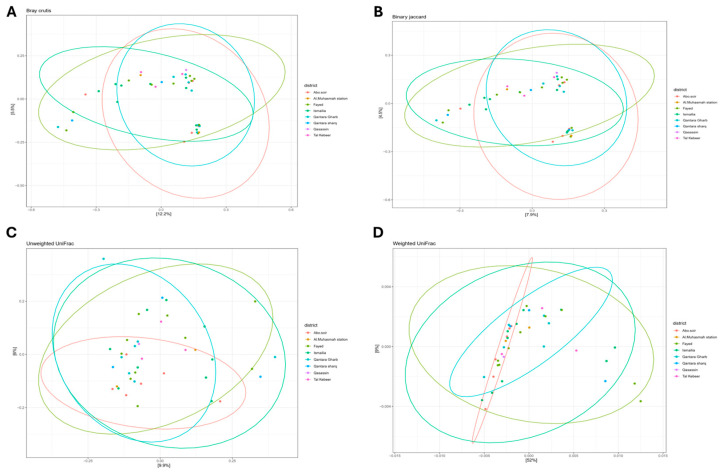
Beta diversity of the raw surface water samples as revealed by Principal Coordinate Analysis (PCoA) using (**A**) Bray–Curtis, (**B**) Binary Jaccard, (**C**) Unweighed Unifarc, and (**D**) Weighed Unifarc dissimilarity metrics according to sampling district. PERMANOVA was used to assess the significance at *p*-value ≤ 0.05. Eclipse denotes significant clustering. Each dot represents one sample.

Beta diversity showed no significant differences in bacterial community structures with respect to sample districts as revealed by Bray–Curtis (*p* = 0.76) and Binary Jaccard (*p* = 0.78) distances (Figure 7). Similarly, there was no significant difference in the phylogenetic relationships of the bacterial community members between these samples as revealed by unweighted UniFrac (*p* = 0.98) and weighted UniFrac (*p* = 0.78) distances (Figure 7). The same was true when assessing the differences in bacterial community composition by PERMANOVA pairwise analysis.

#### 3.3.3. Taxonomic Assignment and Core Bacterial Community

According to BLCA taxonomy, the most predominant phyla were *Proteobacteria* (38.9%) and *Actinobacteriota* (28.8%), followed by *Bacteroidota* (9.6%), *Cyanobacteria* (5.2%), *Verrucomicrobiota* (3.7%), and *Gemmatimonadetes* (0.04%) and *Chlamydiae* (0.01%). On the family level, the most dominant families were *Comamonadaceae* (12.94%), *Intrasporangiaceae* (8.4%), *Ilumatobacteraceae* (7.97%), *Burkholderiaceae* (6.05%), *Streptomycetaceae* (5.02%), followed by *Synechococcaceae* (4.01%), *Rhodobacteraceae* (3.66%), *Chitinophagaceae* (3.3%); the rest of the families showed a relative abundance of less than 3%. On the genus level, the most predominant genera were *Ilumatobacter* (7.97%), *Limnohabitans* (7.26%), *Demequina* (6.34%), *Synechococcus* (4.18%), and *Yinghuangia* (3.77%). The rest showed a relative abundance of less than 3%. On the species level, the most dominant ones were *Terrabacter tumescens* (8.01%), *Ilumatobacter fluminis* (7.97%), *Synechococcus rubescens* (4.18%), *Limnohabitans planktonicus* (4.09%), and *Yinghuangia aomiensis* (3.72%). The rest showed a relative abundance of less than 3%. The overall relative abundances of the top ten phyla, families, genera, and species are shown in Figure S7. Taxa relative abundance in raw surface water samples with respect to the district is shown in Figures S8–S12.

#### 3.3.4. Linear Discriminant Analysis Effect Size (LEfSE)

The analysis of bacterial biomarkers using LEfSE revealed that *Sediminibacterium goheungense* (*p* = 0.02) and *Fluviicola taffensis* (*p* = 0.005) were significantly associated with samples taken from the pollution monitoring points. With respect to the canal, *Chakrabartia godavariana* (*p* = 0.005) and *Cypionkella aquatica* (*p* = 0.02) were significantly associated with samples taken from all canals except the Manayef Canal. With respect to districts, *Chakrabartia godavariana* (*p* = 0.0014) was significantly associated with all districts except the Abo soir and Qassasin districts, which are geographically adjacent to each other.

#### 3.3.5. Taxa Correlation Analysis

We conducted network correlation and Spearman rank algorithm correlation analysis on the BLCA dataset. The correlation cut-off value was adjusted to ± 0.7. The estimated correlation networks on the family, genus, and species networks are shown in Figures S13–S15. While strong negative correlations were detected between taxa of the phyla *Actinobacteria* and *Proteobacteria*, there were strong positive correlations between taxa of the phylum *Actinobacteria* or the phylum *Proteobacteria*. These strong correlations were also detectable between taxa of these two phyla and members of the phylum *Bacteriodota*. On the family level, a strong positive correlation cluster was detected between the family *Intrasporangiaceae* and the families *Streptomycetaceae*, *Ilumatobacteraceae*, and *Demequinaceae.* However, a strong negative correlation was detected between the family *Intrasporangiaceae* and the family *Rhodobacteraceae.* Additionally, a strong negative correlation cluster was detected between the family *Ilumatobacteraceae* and the families *Comamonadaceae* and *Rhodobacteraceae.* Obviously, the positively correlated families *Intrasporangiaceae*, *Streptomycetaceae*, *Ilumatobacteraceae*, and *Demequinaceae* all belong to the phylum *Actinobacteria,* while the negatively correlated families *Comamonadaceae* and *Rhodobacteraceae* belong to the phylum *Proteobacteria.* On the genus level, several strong positive correlation clusters were detectable between the genera *Terrabacter*, *Demequina*, *Tetrasphaera*, *Ilumatobacter,* and *Terrimonas*, and the genera *Aquabacterium*, *Escherichia*, and *Niastella*. Additionally, a strong positive correlation was observed between the genus *Cellvibrio* and the genera *Phenylobacterium*, *Elstera*, and *Reyranella*. The same was true between the genus *Phenylobacterium* and the genera *Malikia* and *Curvibacter*. On the other hand, a strong negative correlation cluster was observed between the genus *Gemmobacter* and the genera *Demequina* and *Yinghuangia*. On the species level, a strong correlation cluster was detected between the most abundant species, *Ilumatobacter fluminis*, *Terrabacter tumescens*, and *Terrimonas lutea*. Whereas a strong negative correlation was observed between *Gemmobacter aestuarii* and *Yinghuangia aomiensis*.

#### 3.3.6. Functional Pathway Prediction

A total of 333 predicted KOs (KEGG Orthologies) were grouped into levels 1, 2, and 3. With respect to level 1 KEGG pathways (Figure S16), the main functional features were related to metabolism (31.7%), followed by environmental information processing (26%), cellular processes (20.2%), genetic information processing (16.9%), human diseases (3.4%), and organismal systems (1.8%). The level 2 KEGG pathway functional features (Figure S17) indicated that the pathways related to global and overview maps, membrane transport, nucleotide metabolism, and cellular community-prokaryotes were the highest, with average abundances of 32.3%, 12.3%, 8.9%, and 5.7%, respectively. On level 3, the top 15 KEGG pathways are shown in Figure S18. Metabolic pathways were the most abundant, followed by the biosynthesis of secondary metabolites, biosynthesis of antibiotics, microbial metabolism in diverse environments, and ABC transporters.

**Figure 8:**
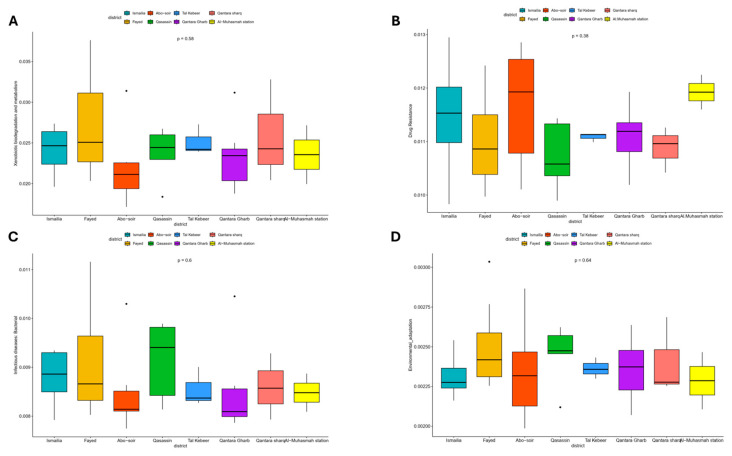
A box plot illustrating the relative abundance of genes related to (**A**) xenobiotic biodegradation, (**B**) antimicrobial drug resistance, (**C**) infectious diseases, and (**D**) environmental adaptation among bacterial communities in raw surface water samples with respect to the sampling district as predicted by the Tax4Fun2 tool. Significant differences between groups were calculated using the Kruskal–Wallis rank sum test. A *p*-value ≤ 0.05 is considered statistically significant. The line inside the box represents the median. Quartiles are box edges. Individual points beyond the whiskers are outliers.

Interestingly, in respect to level 2, KEGG pathways involved in xenobiotic biodegradation, antimicrobial drug resistance, antineoplastic drug resistance, infectious diseases, and environmental adaptation were detected. These were further examined to level 3 KEGG pathways (Figures S19–S22) as they are crucial for ecosystem cleansing and environmental bioremediation. The majority of the xenobiotic degradation activities were related to the role of cytochrome p450 in metabolizing organic pollutants such as benzoate, aminobenzoate, and chloroalkane. Additionally, some metabolic pathways for highly toxic substances like dioxins, atrazine, xylene, and ethylbenzene were also detectable in significant amounts. Bacterial communities of the Fayed, Ismailia, Qasassin, and Qantara Sharq districts showed relatively higher levels of xenobiotic degradation genes compared to other districts, whereas Abo-Soir demonstrated the lowest levels (Figure 8A). However, these differences were statistically non-significant (*p* = 0.58).

Strikingly, Level 3 KEGG pathways related to antimicrobial drug resistance are abundantly involved in beta-lactam resistance, cationic antimicrobial drug resistance, and vancomycin resistance. Bacterial communities of the Abo-Soir district, along with Al-Mahsama station points, showed the highest levels of drug resistance genes (Figure 8B). But these differences were statistically non-significant (*p* = 0.38).

Pathways related to bacterial infections and human disease included level 3 KEGG pathways against legionellosis, which was the most abundant, followed by level 3 KEGG pathways against several pathogens, including *Staphylococcus aureus* infection. Level 3 KEGG pathways related to environmental adaptation are abundantly involved in thermogenesis and plant-pathogen interaction. Bacterial communities of the Qasassin district demonstrated the highest levels of genes related to bacterial infection and environmental adaptation (Figure 8C,D). However, these differences were statistically non-significant (*p* = 0.60 and 0.64, respectively).

Relative Functional Redundancy Index (FRI) values varied across samples. A total of 8826 functions were detected after eliminating the functional features with a zero FRI. These included 472 functions with high FRI, 1158 functions with moderate FRI, and 7196 functions with low FRI. Functions with high FRI values indicate that these functions are used by the vast majority of the bacterial community, whereas functions with low FRI values are more likely to be lost as a result of changes or perturbations in the bacterial community, and suggest that they are only present in a small number of closely related taxa. An FRI value of zero indicates that a function has either not been detected or has only been found in one member of the community.

## 4. Discussion

Changes in the quality of freshwater sources significantly impact the water quality delivered to consumers and influence the choice of processing technology [50]. To ensure safe use of water for potable, agricultural, recreational, or industrial purposes, its quality must be assessed. Seven districts were chosen for water analysis along the Nile River branches in the Ismailia governorate. Selection of sampling locations was performed in accordance with geographical distribution to cover all districts of the Ismailia governorate. The physicochemical features indicated some key variations between sampling districts. In addition, we detected common bacterial indicators of fecal pollution and potential pathogens posing a public health risk. Moreover, taxonomic profiling and functional prediction of microbial communities were performed in order to identify changes in microbial communities that can be attributed to location.

Literature indicates a link between high concentrations of certain physicochemical factors and the proliferation of microbiological populations in water [51,52]. Previous reports suggest that microbial community structure can become more dynamic and is significantly influenced by temperature [53]. In our study, water temperatures consistently exceeded 24 °C, ranging from 24.5 to 28.2 °C. Given these temperatures, a relatively high occurrence and density of coliform bacteria were anticipated. The pH levels ranged from 6.7 to 8.6, which aligns with Egyptian drinking water quality standards [27]. The Nile River typically has a slightly alkaline pH [29,54]. The DO is a crucial indicator of water quality, impacting its suitability for aquatic life and human consumption [12]. In the current study, DO levels were lower than the acceptable permissible limits at sites with high bacterial coliform counts. This finding aligns with our observation of the inverse relationship between BOD and DO; a decrease in DO typically indicates a higher BOD, which is consistent with high bacterial loads [55]. BOD measures the amount of dissolved oxygen required by aerobic microorganisms to decompose the organic carbon of wastewater. High organic matter concentration raises the BOD values [56]. In our study, elevated BOD levels were observed at sites with high bacterial loads, notably at the entrance and exit of the Al-Mahsama wastewater treatment station, besides several sampling points within the Qasassin and Fayed districts. This was consistent with our correlation analysis that showed a positive relationship between BOD and both total and fecal coliform levels. In concordance, raw surface water samples from the Qasassin district demonstrated notably high levels of total and fecal coliforms, and common bacterial contaminants were recovered from all water samples taken from this district. On the other hand, we observed a negative correlation between chloride levels and both total and fecal coliform counts. This is consistent with our observation related to the high chloride levels in the Qantara Sharq district, where raw surface water samples from this location demonstrated low total and fecal coliform levels compared to other locations. This aligns with Aram et al. 2021 who suggested that surface water sources with higher chloride levels are less likely to be contaminated by fecal coliform [57]. In contrast, magnesium levels were notably high at all pollution monitoring points in Qantara Sharq district, particularly in samples from the Al-Abtal Canal. This increase in magnesium concentration may be attributed to the periodic closure of the canal and reopening each month, which could lead to a higher concentration of minerals due to reduced water replenishment. Coliform bacteria are commonly found in the environment and generally do not pose a direct health risk; however, their presence indicates potential contamination by pathogenic microorganisms. Previous studies have shown a significant increase in coliform levels of surface water streams following the addition of sewage outlet points [24,56]. Similarly, our study found the highest coliform load at the exit of Al-Mahsama sewage station.

Fecal coliform bacteria, primarily *E. coli*, and Fecal streptococci are indicators of recent fecal contamination in water systems [58]. Their presence indicates contamination by human or animal feces and, consequently, potential sewage pollution, referring to a higher possibility that fecal-related pathogens are present. In our study, fecal coliforms were present in 80% of the samples, with concentrations ranging from 0 to 1400 MPN/100 mL. Our results are in line with the findings of many other studies reporting comparable levels of total and fecal coliforms in the Nile River [12,19,21,24,59,60]. However, *E. coli* was detected in 50.8% of our samples. This detection rate is higher compared to Azzam et al. 2017 [24], who found *E. coli* in 28.2% of samples from drains and points along the Rosette branch of the Nile. Similarly, Abo-State et al. 2012 [22] reported *E. coli* in 20.1% of their samples. Other studies on the Nile River reported even lower percentages, such as Sabae and Rabeh 2006 [19], who found *E. coli* in 16% of their samples. Conversely, Ali et al. in Aswan, Egypt, reported *E. coli* in all water samples [20]. Fecal streptococci were found in 77.9% of the samples. This is in contrast with some studies that observed lower levels of fecal streptococci, such as 15.6% [24], and a study on the main Nile stream in Sudan, which reported 24% [61]. Other detected bacterial contaminants with potential pathogenic features included *P. aeruginosa* and *S. aureus.* The bacterium *P. aeruginosa* was found in 49.1% of samples, which is comparable to a study on Nile water in Sudan [61], which reported a 38% prevalence. However, other studies reported lower levels of *P. aeruginosa* at 19.3% [24] and 12% [19]. The *S. aureus* was detected in 77.9% of samples, which aligns with Goja et al. 2013 [61], who reported high levels of *S. aureus* at 62.4%. In contrast, other studies [19,24] detected *S. aureus* with percentages of 14.1% and 16%, respectively. Strikingly, *E. coli*, fecal streptococci, *P. aeruginosa,* and *S. aureus* were recovered from all water samples of the Qasassin district, posing a public health risk in this area and harming to freshwater ecosystem.

Over 99% of microbes in many environments cannot be cultured [16]. High-throughput DNA sequencing provides a means to characterize these uncultured microbes in their natural habitats [62], enabling a comprehensive understanding of the biological diversity present in water samples, from groundwater to drinking water [63]. In the current study, we used 16S rRNA sequencing technology to explore bacterial strains within the freshwater microbiome. Based on alpha and beta diversity indices, there was no significant difference in bacterial diversity within samples between the investigated sampling locations. There was also no significant difference in bacterial community structures between different sampling locations, indicating resilience and stability of the raw surface water microbiome. This was in concordance with Eraqi and colleagues, who reported high resilience and stability of the Nile River microbiome in Cairo [57]. Generally, the dominant phyla identified in the present study included *Proteobacteria*, *Actinobacteria*, *Bacteroidetes*, *Cyanobacteria*, and *Verrucomicrobia*. All these were previously reported as characteristic members of freshwater environments [18,64,65,66]. In consistent with Eraqi 2018 [25], who investigated the microbial communities of the River Nile in Cairo, *Proteobacteria* and *Actinobacteriota* were the most predominant phyla. In addition, earlier studies indicated *Actinobacteria* and *Proteobacteria* as highly abundant members in river ecosystems [67]. The most dominant species within the *Actinobacteria* phylum in our study were *Ilumatobacter fluminis* and *Terrabacter tumescens*, which are both common soil inhabitants. Remarkably, these species were also the top two species across the entire microbiome analyzed. They are known for their ability to utilize a wide range of organic compounds and their capability to reduce nitrate to nitrite and ammonia [68,69]. These traits further support the presence of organic matter in our water samples, as indicated by the elevated BOD levels found in our study. On the contrary, Yosef et al. 2022 [70] found *Actinobacteria* comprising only 5% of the microbial community in the hypersaline Lake Qarun, indicating different microbial community profiles with respect to the nature of the water ecosystem. *Cyanobacteria*, such as *Synechococcus*, are known for their adaptability to a wide range of water sources, including those that are contaminated or polluted [71]. In our study, *Cyanobacteria* were exclusively categorized as *Synechococcus rubescens* species. In consistency, this species was identified as a dominant member of the *Cyanobacteria* phylum in East Fork Lake (87.9%) and Delaware Lake (73.5%) in a study on beach waters in Ohio [72].

Network-based methods have been successful in understanding complex microbial interaction patterns using microbiome profiling data [73]. Barberán et al. 2021 [46] suggest that habitat network structures may reflect ecological principles that shape microbial communities. We observed that within the *Actinobacteria* phyla, almost all species showed positive correlations with each other, suggesting a high level of community cohesion. Interestingly, there was a negative correlation observed between genera belonging to *Actinobacteria* and those belonging to *Proteobacteria*. This negative correlation suggests that these two groups may occupy different ecological niches or compete for similar resources within the microbial community, leading to an inverse relationship in their abundances. This kind of interaction highlights the complex dynamics within microbial ecosystems, where competition and cooperation shape community structure and function.

Functional prediction offers valuable insights into critical aspects of an ecosystem microbiome that impact public health, such as pathogenicity and antibiotic resistance. Although shotgun metagenomic sequencing is the gold standard for functional prediction [74], the Tax4Fun tool, which we used in the current study, is a valuable alternative based on 16S rRNA sequencing. This approach offers a reliable approximation of functional profiles typically obtained through metagenomic shotgun sequencing [75]. Understanding the link between microbial systems and ecosystem processes is a crucial point of view because it is assumed that environmental microbes are functionally redundant [76]. A more comprehensive understanding of the role of biodiversity in maintaining ecosystem function can be obtained by evaluating multiple bacterial functions [77]. In accordance with others [70], pathways relating to metabolism were the most predominant level 1 functional features among our bacterial communities, followed by cellular functions, genetic information processing, human diseases, and organismal systems. Further analysis for more detailed functional features on levels 2 and 3 detected several pathways related to xenobiotic biodegradation, antimicrobial drug resistance, antineoplastic drug resistance, human infectious diseases, and environmental adaptation. These are among the most interesting functional abilities of bacterial microbiomes, in particular xenobiotic biodegradation, which may aid the ecosystem in cleaning up or bioremediating the environment, particularly in relation to water quality, and may also contribute to the degradation of anthropogenic pollutants, mitigating potential harm to animals and humans [78]. However, these functions are also of particular concern for public health, posed by the emerging antimicrobial resistance [74]. We observed relatively high levels of xenobiotic biodegradation genes in water microbial communities of the Fayed, Ismailia, Qasassin, and Qantara Sharq districts, whereas water microbial communities of the Abo-Soir district, along with Al-Mahsama wastewater treatment station, demonstrated relatively high levels of drug resistance genes. Antibiotic resistance level was previously linked to specific traits of microbial communities, including pathogenic phenotypes and xenobiotic biodegradation [74]. Previous studies on the Nile water show an increase in antibiotic resistance because of the extensive use and misuse of antibiotics [24]. Moreover, despite having fewer samples compared to other districts, water microbial communities of the Qassasin district demonstrated the highest levels of bacterial infectious disease genes. This finding corroborates our culture results of total and fecal coliforms and detection of common bacterial contaminants, which pose a heightened risk for waterborne diseases in this area. However, although Tax4Fun is a practical tool, recent research highlights that functional inference using 16S rRNA data is fundamentally constrained by both methodological and biological factors, especially in accurately reflecting the metabolic potential at the community level. The accuracy of these predictions heavily depends on how well taxa are represented in reference genome databases. In complex environmental microbiomes, where many organisms are unclassified or poorly represented, the predicted functions might be incomplete or systematically skewed [47,79,80]. Additionally, since the 16S rRNA gene is a phylogenetic marker rather than a functional one, Tax4Fun2 cannot detect strain-level genomic variations, horizontally transferred genes, or new metabolic pathways that differ from taxonomic predictions [47,79,80]. Therefore, while Tax4Fun2 is a valuable tool for exploration and hypothesis generation, its findings should be approached with caution and, when feasible, confirmed through metagenomic or metatranscriptomic studies.

A central question in microbial ecology is the extent to which FRI exists within microbial communities and its potential to stabilize ecosystem processes in response to disturbances [81]. FRI has been positively correlated with ecological stability and resilience [82]. In accordance with Wemheuer et al. 2020 [47], FRI results varied among our samples, indicating shifting levels of functional redundancy. Research has shown that the variability in functional potentials, as indicated by metagenomic gene abundances, often exceeds that of microbial taxonomic compositions across different ecosystems [76]. Although a significant number of functions displayed high functional redundancy, 472 in total, most of the functional features identified in our study had low relative functional redundancy. This suggests that these functions are predominantly found in a limited number of closely related species, highlighting a lack of functional redundancy across the broader microbial community.

## 5. Conclusions

The present study underscores the importance of continuous monitoring of freshwater resources. Predominant water contaminants included *E. coli* (50.8%), fecal streptococci (79.7%), *Pseudomonas aeruginosa* (49.2%), and *Staphylococcus aureus* (76.3%). Our findings highlight significant microbial contamination, with levels of 100% and 80% of total and fecal coliforms. Physico-chemical and bacteriological analyses linked environmental conditions, such as elevated BOD levels and low DO values, to bacterial contamination, particularly in the Qasassin district. Metagenomic analysis revealed that most samples had similar bacterial community structures, despite location-driven variability. However, functional prediction demonstrated a relatively high level of bacterial infectious genes among water samples from the Qasassin district. The observed risk of water-borne illnesses in this area highlights the need for additional research and ongoing surveillance to protect public health. Our findings underscore the urgent need for integrated water quality management and highlight the value of metagenomic approaches for monitoring microbial health risks in freshwater ecosystems. Future studies should focus on continuous monitoring of freshwater sources to mitigate emerging environmental and public health risks.

## Data Availability

Data available in a publicly accessible repository. The data presented in this study are openly available in [NCBI database] [http://www.ncbi.nlm.nih.gov/bioproject/1074353] (accessed on 7 February 2024) [PRJNA1074353].

## Supplementary Materials

The following supporting information can be downloaded at https://www.mdpi.com/article/10.3390/microorganisms13102395/s1. Figure S1: A map showing the main branches of the Ismailia Canal. At Ismailia, the Ismailia Canal bifurcates into two arms: one to the North to supply Port-Said passing by Qantara-Gharb district (Port-Said Canal), and the second to the South to supply Suez passing by Fayed district (Suez Canal); Figure S2: A map showing the Manayef Canal and the Sinai Canal. There is a small branch of the Ismailia Canal called the Manayef Canal, which runs parallel to it to the Manayef district. In addition, there is a linkage canal connecting both the Ismailia Canal and the Suez Canal, known as the Sinai Canal. Both the Sinai and Suez canals branch off the Ismailia Canal and lead to the South of Ismailia Governorate; Figure S3: A map showing Al-Mahsama sewage treatment station and Al-Abtal Canal on the East side of the Suez Canal. A large wastewater treatment plant, the Al-Mahsama station, was constructed in 2020 in the Sinai Peninsula’s Eastern Suez Canal region to collect, treat, and transfer agricultural drainage water through the Serapeum siphon under the New Suez Canal to Al-Abtal Canal on the Eastern side of the Suez Canal. Al-Abtal Canal ends in the Huda Canal in Qantara Sharq, for agricultural use in Sinai; Figure S4: Agarose gel electrophoresis of the PCR amplicons using primer sequences targeting the 16S rRNA V3–V4 region, including PCR reagent-only extraction control and a PCR no template control alongside a 100 bp DNA ladder. Lanes 1–13 represent amplicons of the 16S rRNA V3–V4 region of raw water samples; Figure S5: Presumptive detection and isolation of common bacterial contaminants. Raw water samples were screened for the presumptive detection and isolation of *P. aeruginosa*, *S. aureus*, *E. coli,* and fecal streptococci. A, Cetrimide agar showing colonies with green pigmentation confirmed the presence and isolation of *P. aeruginosa.* B, Mannitol Salt agar showing yellow colonies with yellow zones confirming the presence and isolation of *S. aureus.* C, Eosin Methylene Blue agar showing colonies with a metallic green sheen, confirming the presence and isolation of *E. coli*. D, Bile Esculin Azide agar showing brownish-black colonies with brown halos, confirming the presence and isolation of fecal streptococci. All isolates were subjected to further identification using the Automated ID and AST System MA120 (Render Biotech Co., Shenzhen, China). The MA120 System functions through integrated colorimetric and turbidimetric methodologies to achieve reliable microbial identification by utilizing biochemical reaction-based color changes; Figure S6: Rarefaction curve. Samples were rarefied to a sampling depth of 5500. The library size ranged from 2506 to 19461 reads, with an average of 8957 reads per sample; Figure S7: The overall relative abundances of the top taxa in raw water samples in respect to (A) phyla, (B) families, (C) genera, and (D) species; Figure S8: Grid bar charts showing the relative abundance of the most predominant phyla in raw water samples in respect to district in the following order: Abo-soir, Al-Mahsama station, Fayed, Ismailia, Qantara Gharb, Qantara Sharq, Qasassin, and Tal-kabeer; Figure S9: Grid bar charts showing the relative abundance of the most predominant families in raw water samples in respect to district in the following order: Abo-soir, Al-Mahsama station, Fayed, Ismailia, Qantara Gharb, Qantara Sharq, Qasassin, and Tal-kabeer; Figure S10: Grid bar charts showing the relative abundance of the most predominant genera in raw water samples in respect to district in the following order: Abo-soir, Al-Mahsama station, Fayed, Ismailia, Qantara Gharb, Qantara Sharq, Qasassin, and Tal-kabeer; Figure S11: Grid bar charts showing the relative abundance of the most predominant species in raw water samples in respect to district in the following order: Abo-soir, Al-Mahsama station, Fayed, Ismailia, Qantara Gharb, Qantara Sharq, Qasassin, and Tal-kabeer; Figure S12: Taxa relative abundance in raw water samples in respect to district in the following order: Abo-soir, Al-Mahsama station, Fayed, Ismailia, Qantara Gharb, Qantara Sharq, Qasassin, and Tal-kabeer. Stacked bar charts show the relative abundance of the most predominant (A) phyla, (B) families, (C) genera, (D) species of the water microbiome in raw water samples. Each bar represents one sample; Figure S13: Correlation network based on estimates of taxa co-occurrence at the family level in raw water samples. The size of each node represents the relative abundance of the corresponding taxa, and the node color indicates the ancestor phylum. Red edges represent a positive correlation, while blue edges represent a negative correlation. The correlation cut-off value was adjusted to ±0.7; Figure S14: Correlation network based on estimates of taxa co-occurrence at genus level in raw water samples. The size of each node represents the relative abundance of the corresponding taxa, and the node color indicates the ancestor phylum. Red edges represent a positive correlation, while blue edges represent a negative correlation. The correlation cut-off value was adjusted to ±0.7; Figure S15: Correlation network based on estimates of taxa co-occurrence at the species level in raw water samples. The size of each node represents the relative abundance of the corresponding taxa, and the node color indicates the ancestor phylum. Red edges represent a positive correlation, while blue edges represent a negative correlation. The correlation cut-off value was adjusted to ±0.7; Figure S16: The relative abundance of the primary functional features among bacterial communities in water samples on level 1 as predicted by Tax4Fun2 tool; Figure S17: The relative abundance of the primary functional features among bacterial communities in water samples on level 2 and their corresponding functional features on level 1, as predicted by Tax4Fun2 tool; Figure S18: The overall relative abundance of the top 10 functional features among bacterial communities in water samples on level 3 and their corresponding functional features on level 1 and 2, as predicted by Tax4Fun2 tool; Figure S19: The relative abundance of level 3 KEGG pathways belonging to xenobiotic biodegradation and metabolism and their corresponding functional features on level 1, as predicted by Tax4Fun2 tool; Figure S20: The relative abundance of level 3 KEGG pathways belonging to drug resistance and their corresponding functional features on level 1, as predicted by Tax4Fun2 tool; Figure S21: The relative abundance of level 3 KEGG pathways belonging to bacterial infectious diseases and their corresponding functional features on level 1, as predicted by Tax4Fun2 tool; Figure S22: The relative abundance of level 3 KEGG pathways belonging to environmental adaptation and their corresponding functional features on level 1, as predicted by Tax4Fun2 tool; Table S1: Summary of collected raw water samples; Table S2: Physicochemical properties of raw water samples.

## Abbreviations

The following abbreviations are used in this manuscript:

TDS	Total dissolved solids
BOD	Biochemical oxygen demand
DO	Dissolved oxygen
NH_3_	Ammonia
NO_3_	Nitrate
NO_2_	Nitrite
Cl	Chloride
CaH	Calcium hardness
MgH	Magnesium hardness
Ca	Calcium
Mg	Magnesium
TH	Total hardness
T.Alk	Total alkalinity
QIIME 2	Quantitative Insights into Microbial Ecology 2
DADA 2	Divisive Amplicon Denoising Algorithm 2
ASV	Amplicon Sequence Variants
BH	Benjamini–Hochberg
PCoA	Principal Coordinate Analysis
PERMANOVA	Permutation-based ANOVA
BLCA	Bayesian Least Common Ancestor
LEfSE	Linear discriminant analysis effect size
LDA	linear discriminant analysis
KO	KEGG Orthology
FRI	Functional Redundancy Index
