# Supplementary material for: Microbial Communities and Physicochemical Properties of the Nile River Water in the Suez Canal Area
Source: Microorganisms. 2025 Oct 19;13(10):2395. doi: 10.3390/microorganisms13102395 (PMC12566090; doi:10.3390/microorganisms13102395)
Supplement: Supplementary file 1 [file microorganisms-13-02395-s001.zip › microorganisms-3896096-supplementary.pdf]

Title: Microbial communities and physicochemical properties of the Nile River water in the Suez Canal area

Noha Elkayal[1], Samira Zakeer[1], Marwa Azab[1], Ali Abdellah[1], Sarah Shabayek[1*]

[1] Department of Microbiology and Immunology, Faculty of Pharmacy, Suez Canal University, Ismailia, 41522 Egypt.

* Corresponding author: Sarah Shabayek, Microbiology and Immunology Department, Faculty of Pharmacy, Suez Canal University, Ismailia, 41522 Egypt

* **Correspondence:** Sarah Shabayek**,**

Postal address: Department of Microbiology and Immunology, Faculty of Pharmacy, Suez Canal University, 41522 Ismailia, Egypt

Email address: sarah.shabayek@pharm.suez.edu.eg

ORCID ID: https://orcid.org/0000-0001-8218-674X

Institutional phone number: 002-064-32001258

1. Supplementary Figures:

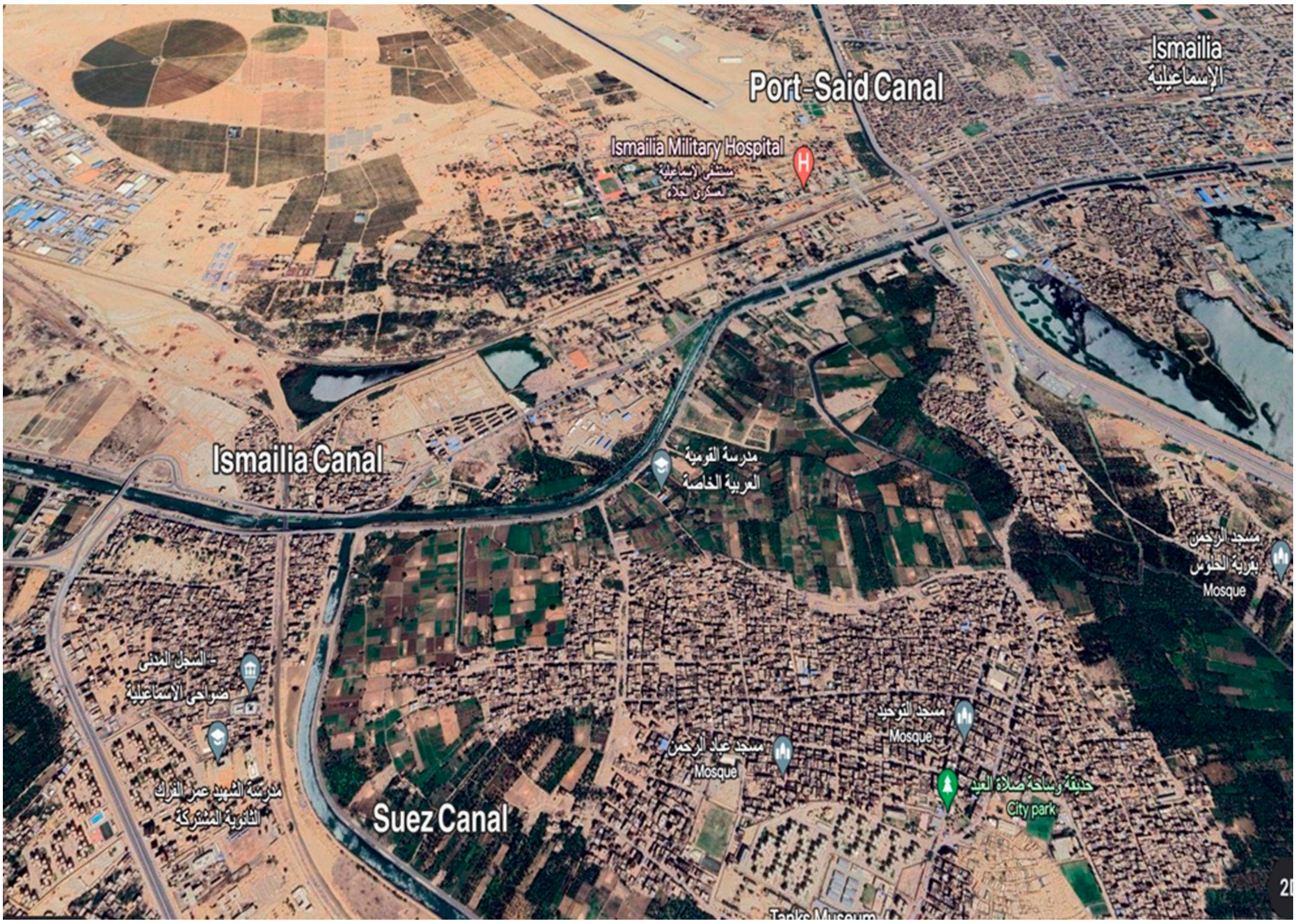

Figure S1. A map showing the main branches of the Ismailia Canal. At Ismailia, the Ismailia Canal bifurcates into two arms: one to the North to supply Port-Said passing by Qantara-Gharb district (Port-Said Canal), and the second to the South to supply Suez passing by Fayed district (Suez Canal).

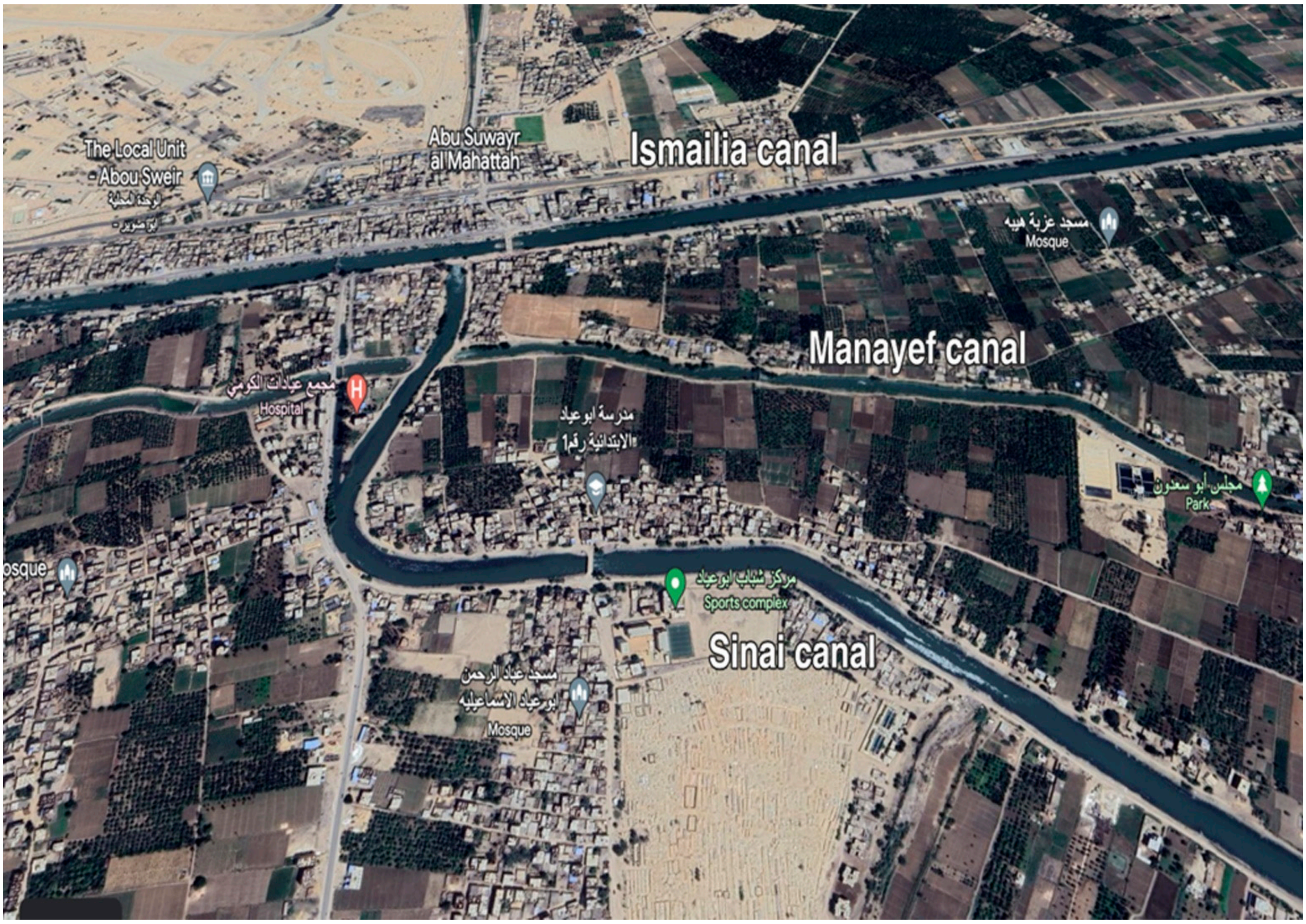

Figure S2. A map showing the Manayef Canal and the Sinai Canal. There is a small branch of the Ismailia Canal called the Manayef Canal which runs parallel to it to the Manayef district. In addition, there is a linkage canal connecting both the Ismailia Canal and Suez Canal known as the Sinai Canal. Both Sinai and Suez canals branch off the Ismailia Canal and lead to the South of Ismailia Governorate

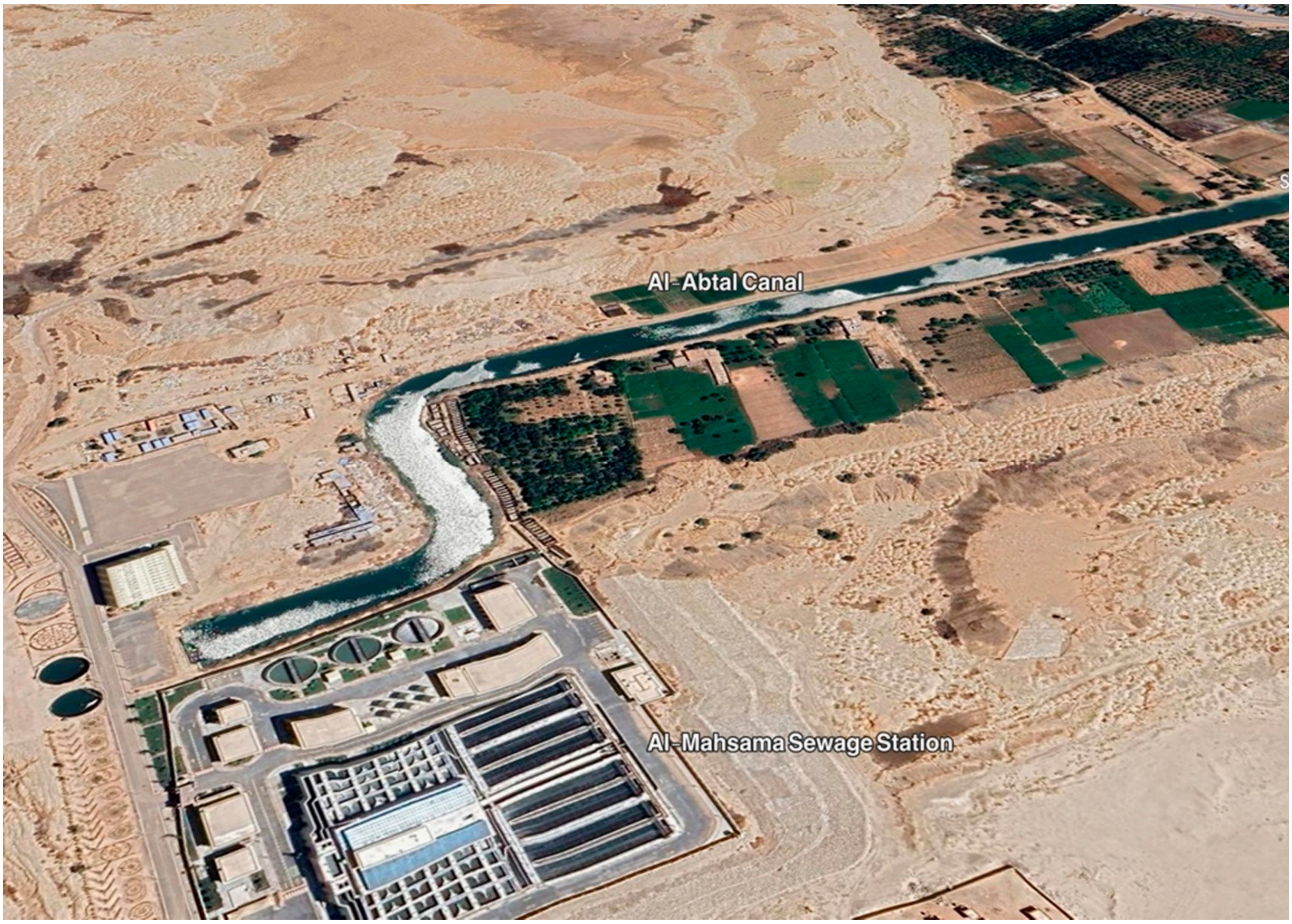

Figure S3. A map showing Al-Mahsama sewage treatment station and Al-Abtal Canal on the East side of the Suez Canal. A large wastewater treatment plant, the Al-Mahsama station, was constructed in 2020 in the Sinai Peninsula's Eastern Suez Canal region to collect, treat, and transfer agricultural drainage water through the Serapeum siphon under the New Suez Canal to Al-Abtal Canal on the Eastern side of the Suez Canal. Al-Abtal Canal ends in the Huda Canal in Qantara Sharq, for agriculture use in Sinai

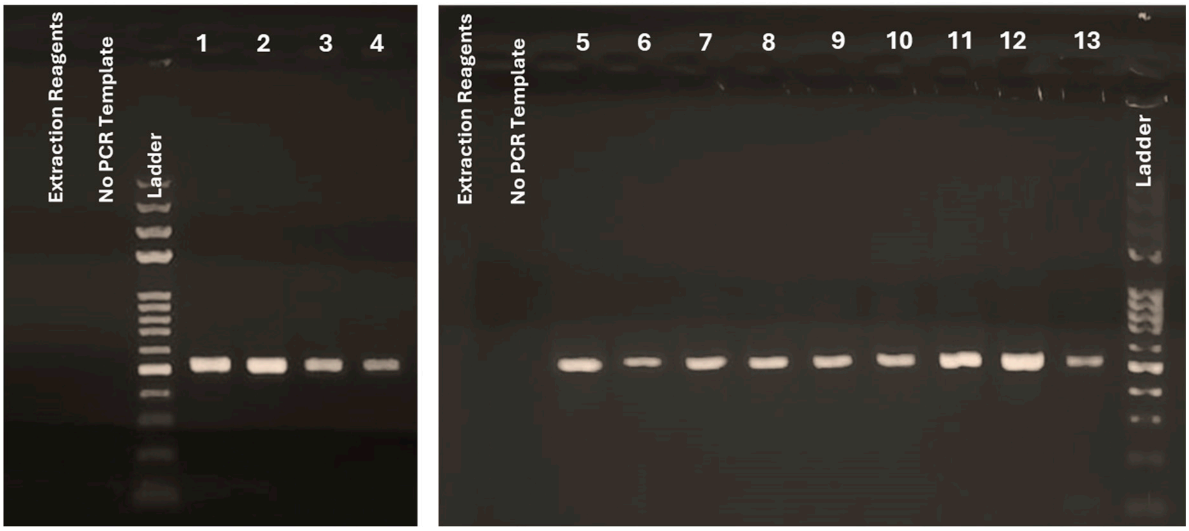

Figure S4. Agarose gel electrophoresis of the PCR amplicons using primer sequences targeting the 16S rRNA V3 - V4 region including PCR reagent-only extraction control and a PCR no template control alongside a 100 bp DNA ladder. Lanes 1 - 13 represent amplicons of the 16S rRNA V3 - V4 region of raw water samples.

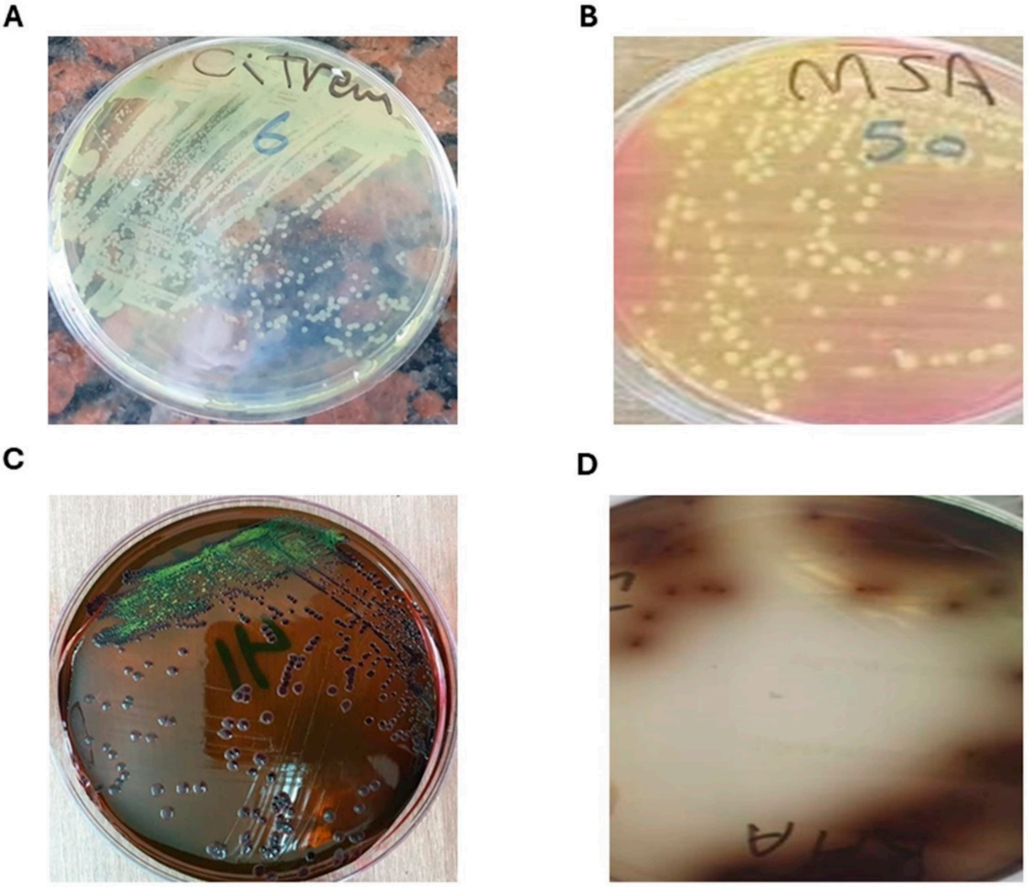

Figure S5. Presumptive detection and isolation of common bacterial contaminants. Raw surface water samples were screened for the presumptive detection and isolation of *P. aeruginosa, S. aureus, E. coli* and fecal streptococci. A, Cetrimide agar showing colonies with green pigmentation confirmed the presence and isolation of *P. aeruginosa.* B, Mannitol Salt agar showing yellow colonies with yellow zones confirming the presence and isolation of *S. aureus.* C, Eosin Methylene Blue agar showing colonies with a metallic green sheen confirming the presence and isolation of *E. coli*. D, Bile Esculin Azide agar showing brownish-black colonies with brown halos confirming the presence and isolation of fecal streptococci. All isolates were subjected to further identification using Automated ID & AST System MA120 (Render Biotech Co., Shenzhen, China). The MA120 System functions through integrated colorimetric and turbidimetric methodologies to achieve reliable microbial identification by utilizing biochemical reaction-based color changes.

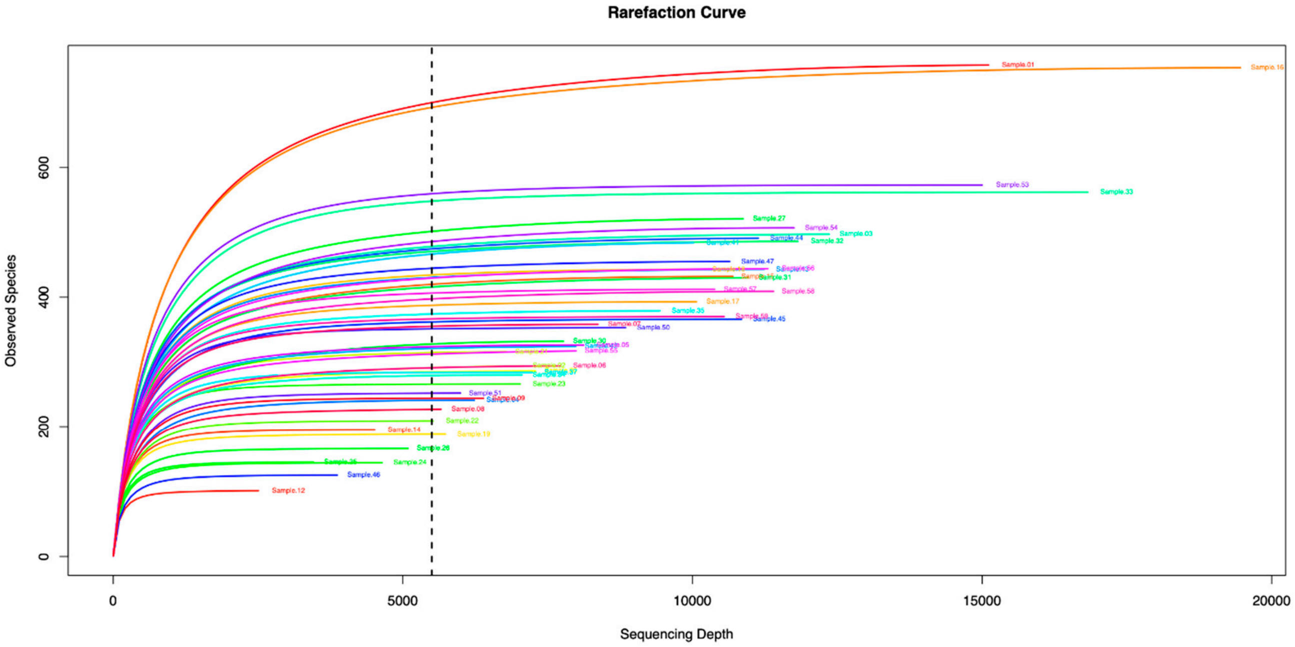

Figure S6. Rarefaction curve. Samples were rarefied to a sampling depth of 5500. The library size ranged from 2506 to 19461 reads, with an average of 8957 reads per sample.

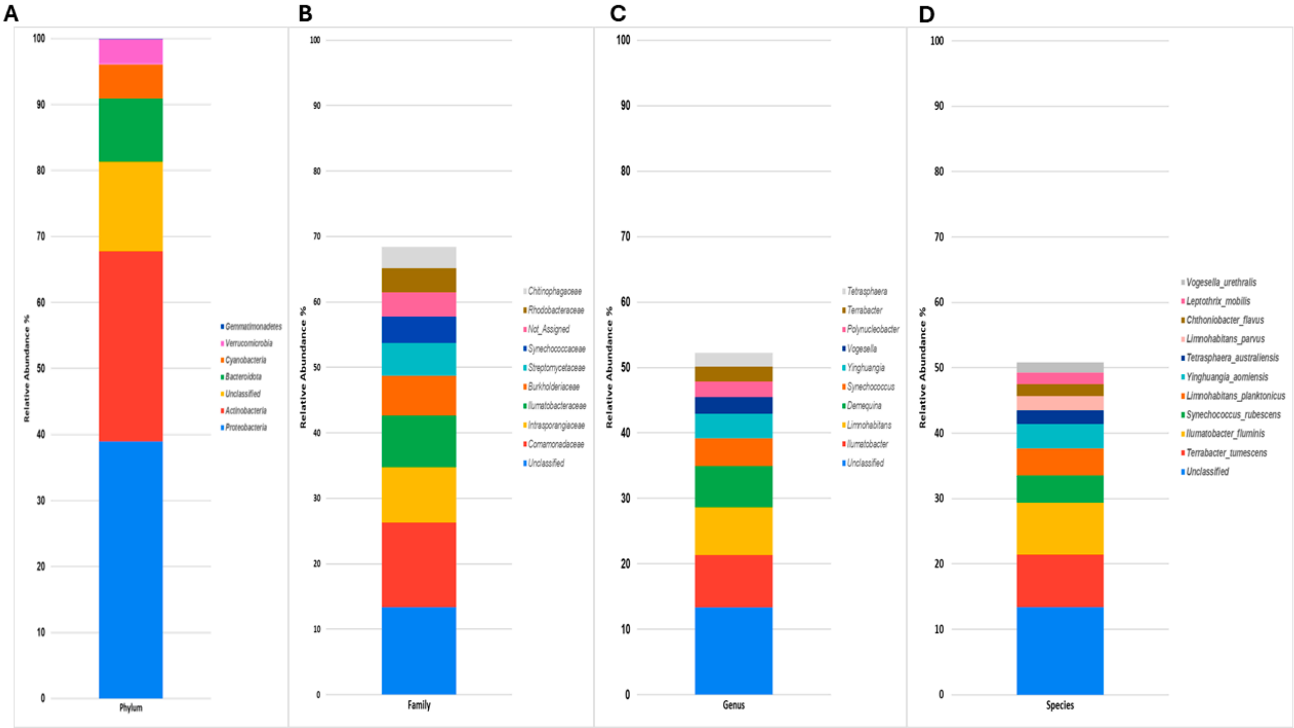

Figure S7. The relative abundances of the top taxa in raw surface water samples in respect to (A) phyla, (B) families, (C) genera, and (D) species.

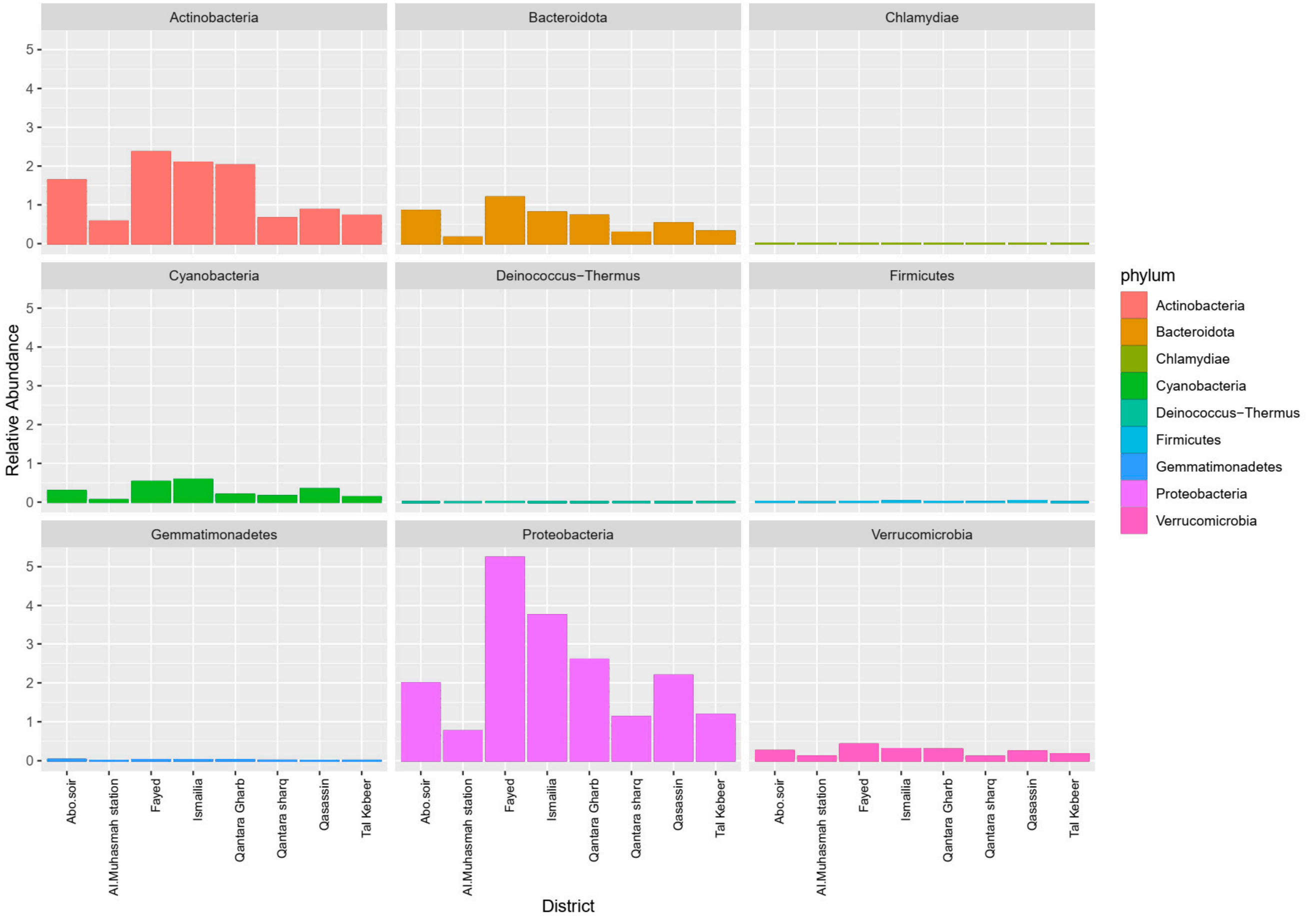

Figure S8. Grid bar charts showing the relative abundance of the most predominant phyla in raw surface water samples in respect to district in the following order: Abo-soir, Al-Mahsama station, Fayed, Ismailia, Qantara Gharb, Qantara Sharq, Qasassin, and Tal-kabeer.

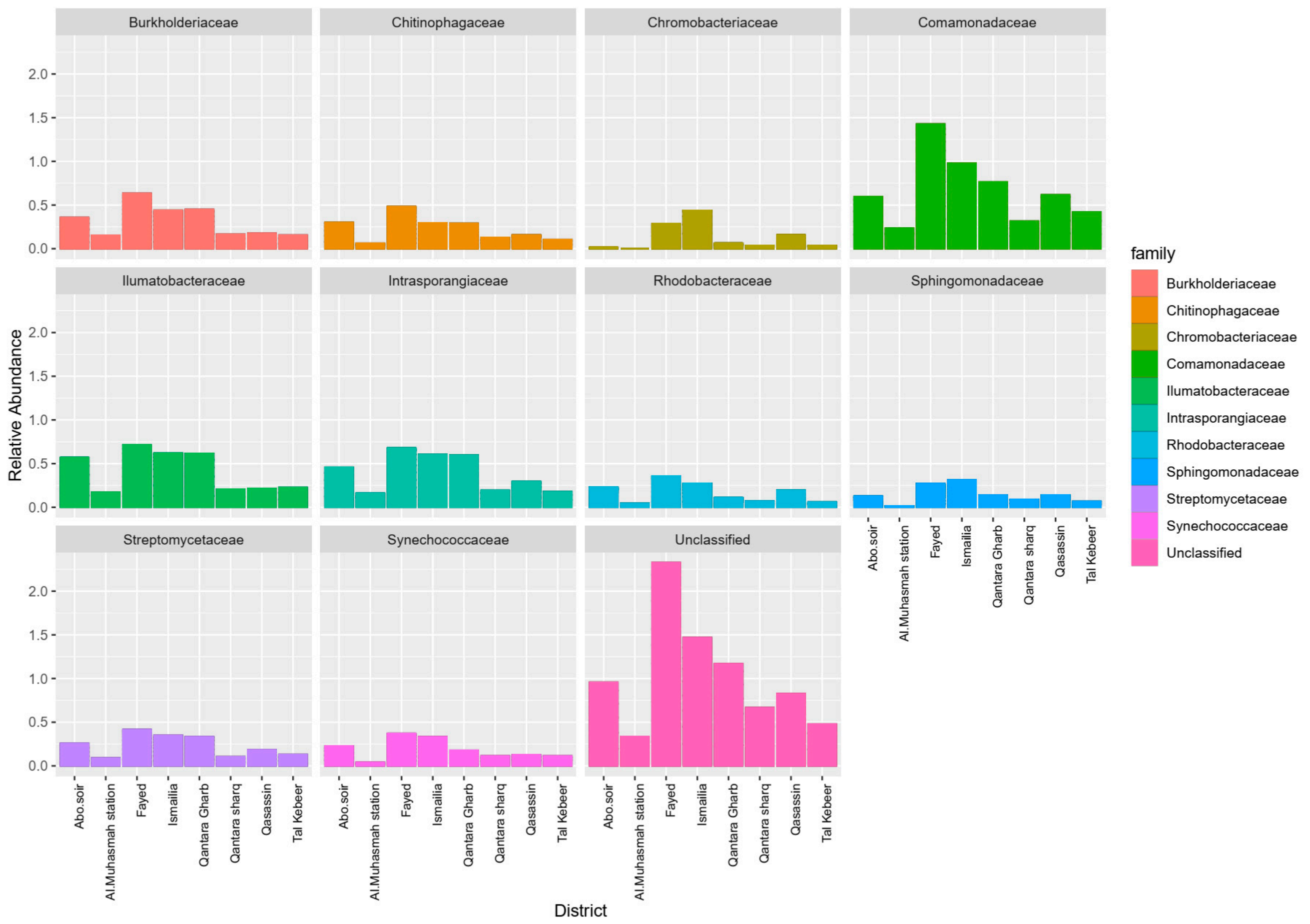

Figure S9. Grid bar charts showing the relative abundance of the most predominant families in raw surface water samples in respect to district in the following order: Abo-soir, Al-Mahsama station, Fayed, Ismailia, Qantara Gharb, Qantara Sharq, Qasassin, and Tal-kabeer.

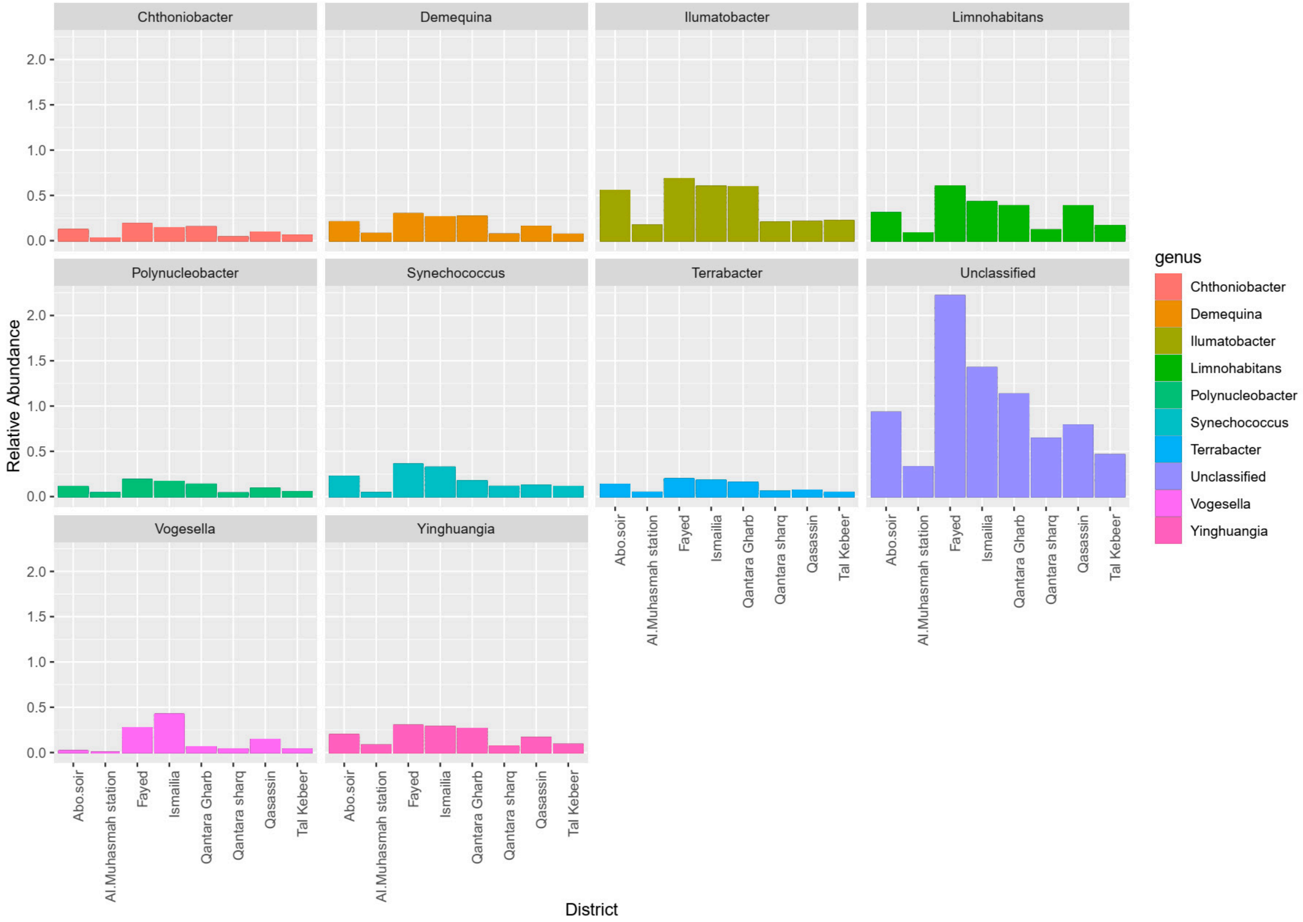

Figure S10. Grid bar charts showing the relative abundance of the most predominant genera in raw surface water samples in respect to district in the following order: Abo-soir, Al-Mahsama station, Fayed, Ismailia, Qantara Gharb, Qantara Sharq, Qasassin, and Tal-kabeer.

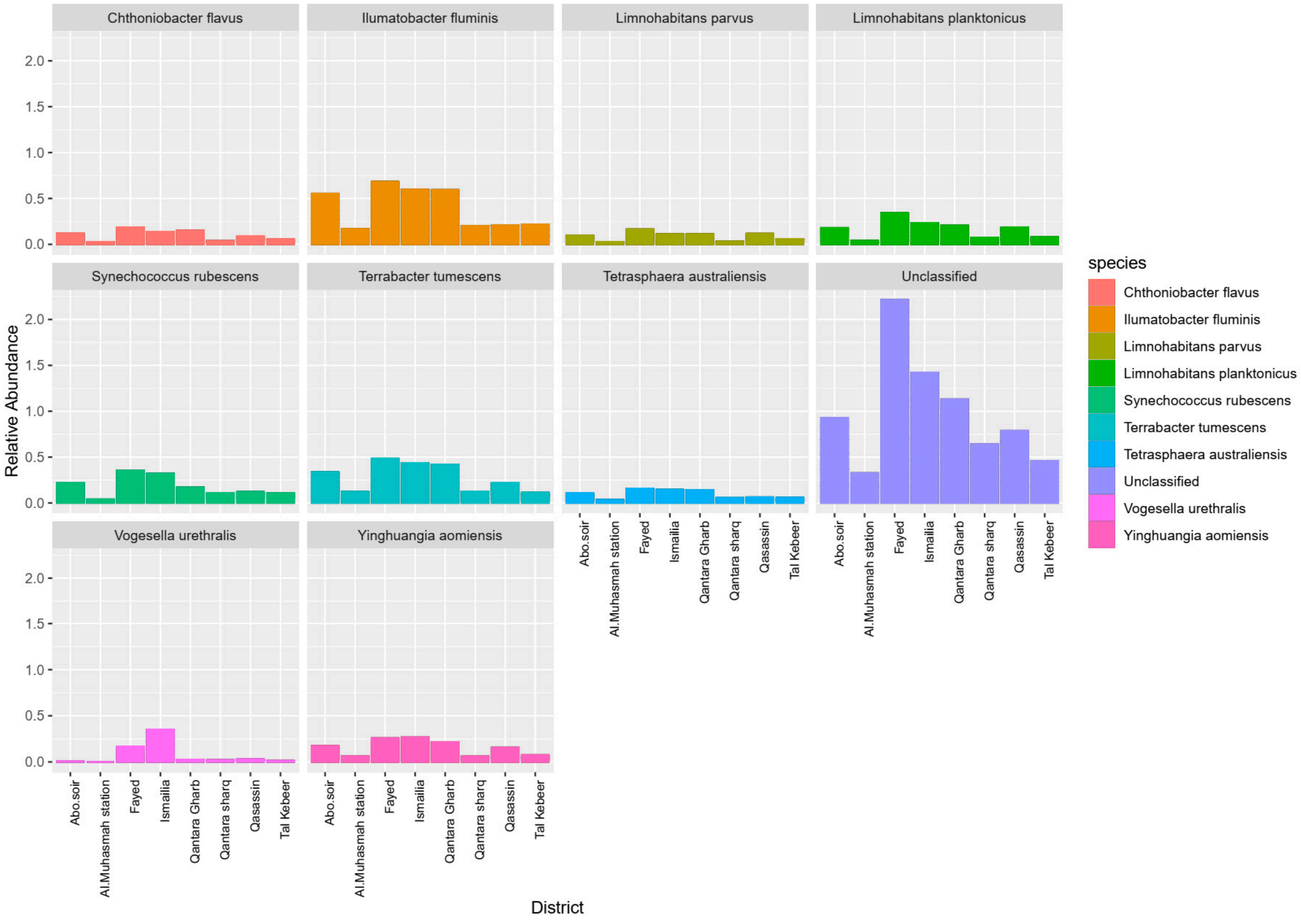

Figure S11. Grid bar charts showing the relative abundance of the most predominant species in raw surface water samples in respect to district in the following order: Abo-soir, Al-Mahsama station, Fayed, Ismailia, Qantara Gharb, Qantara Sharq, Qasassin, and Tal-kabeer.

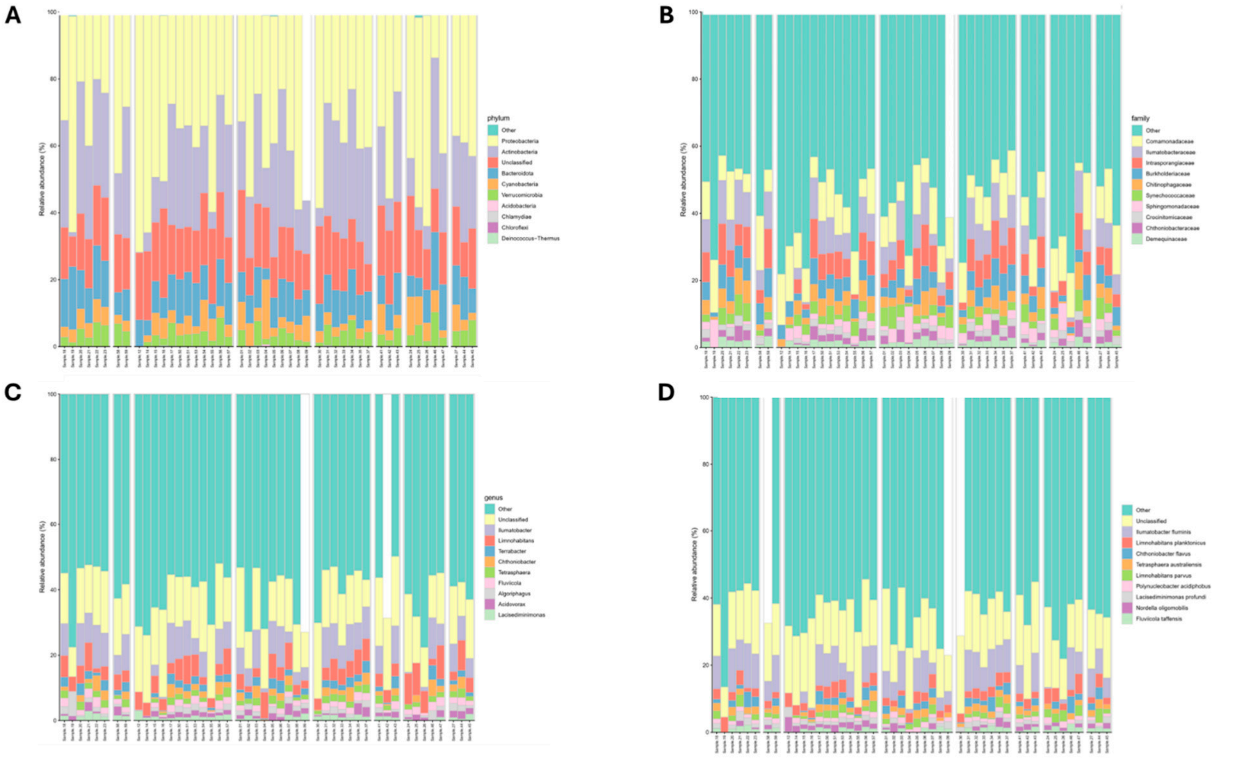

Figure S12. Taxa relative abundance in raw surface water samples in respect to district in the following order: Abo-soir, Al-Mahsama station, Fayed, Ismailia, Qantara Gharb, Qantara Sharq, Qasassin, and Tal-kabeer. Stacked bar charts show the relative abundance of the most predominant (A) phyla, (B) families, (C) genera, (D) species of the water microbiome in raw water samples. Each bar represents one sample.

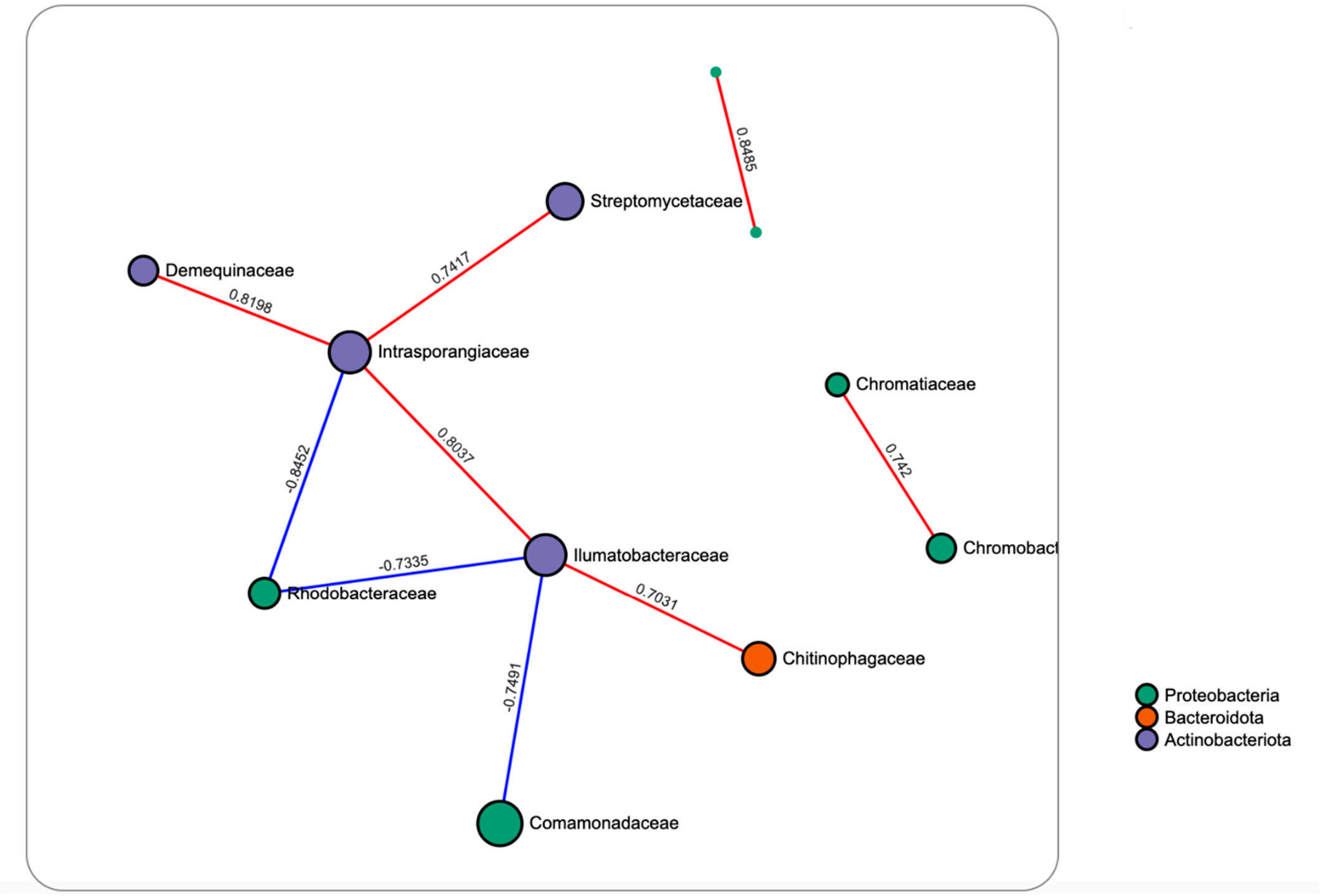

Figure S13. Correlation network based on estimates of taxa co-occurrence at family level in raw surface water samples. The size of each node represents the relative abundance of the corresponding taxa, and the node color indicates the ancestor phylum. Red edges represent a positive correlation, while blue edges represent a negative correlation. The correlation cut-off value was adjusted to ± 0.7.

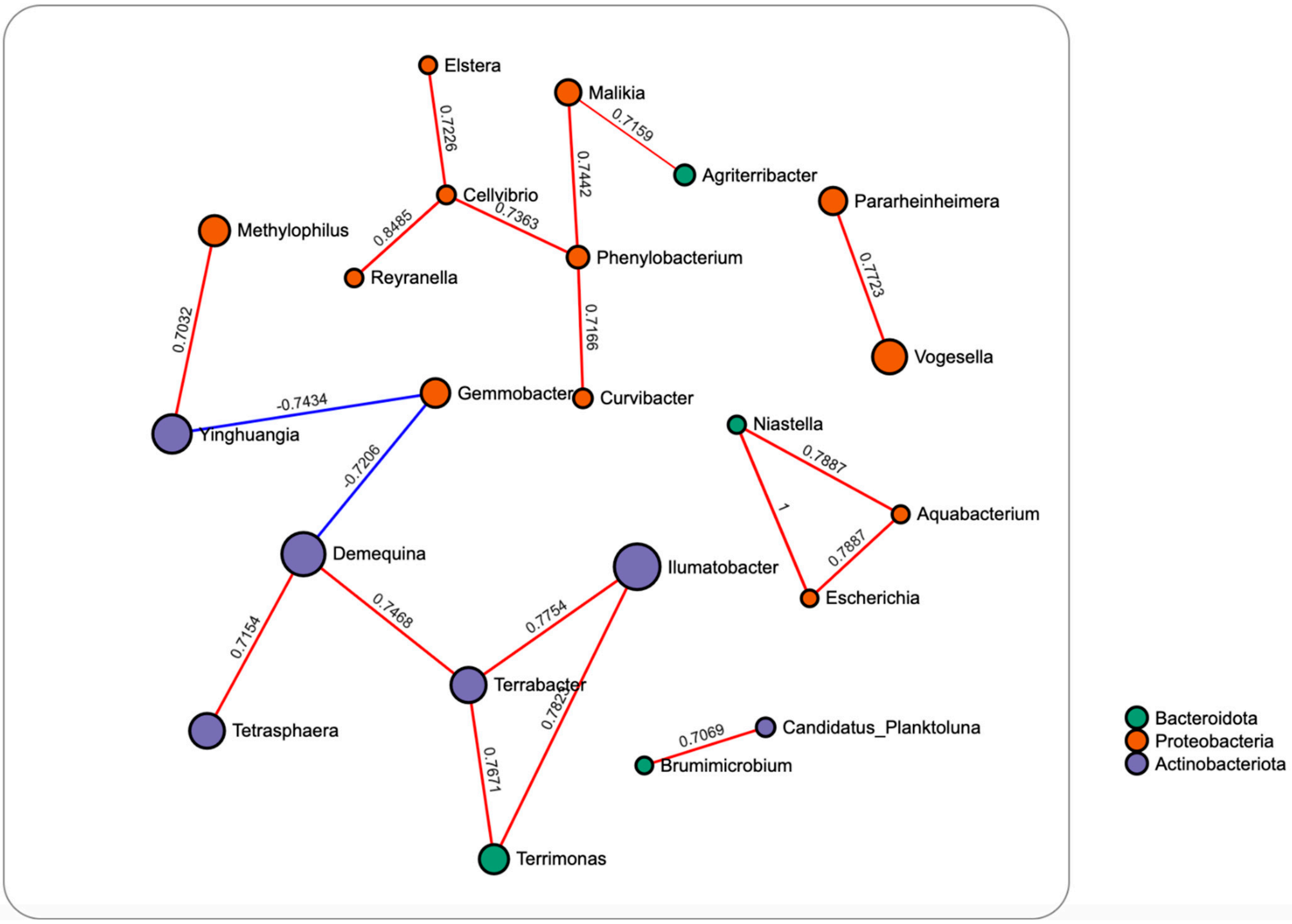

Figure S14. Correlation network based on estimates of taxa co-occurrence at genus level in raw surface water samples. The size of each node represents the relative abundance of the corresponding taxa, and the node color indicates the ancestor phylum. Red edges represent a positive correlation, while blue edges represent a negative correlation. The correlation cut-off value was adjusted to ± 0.7.

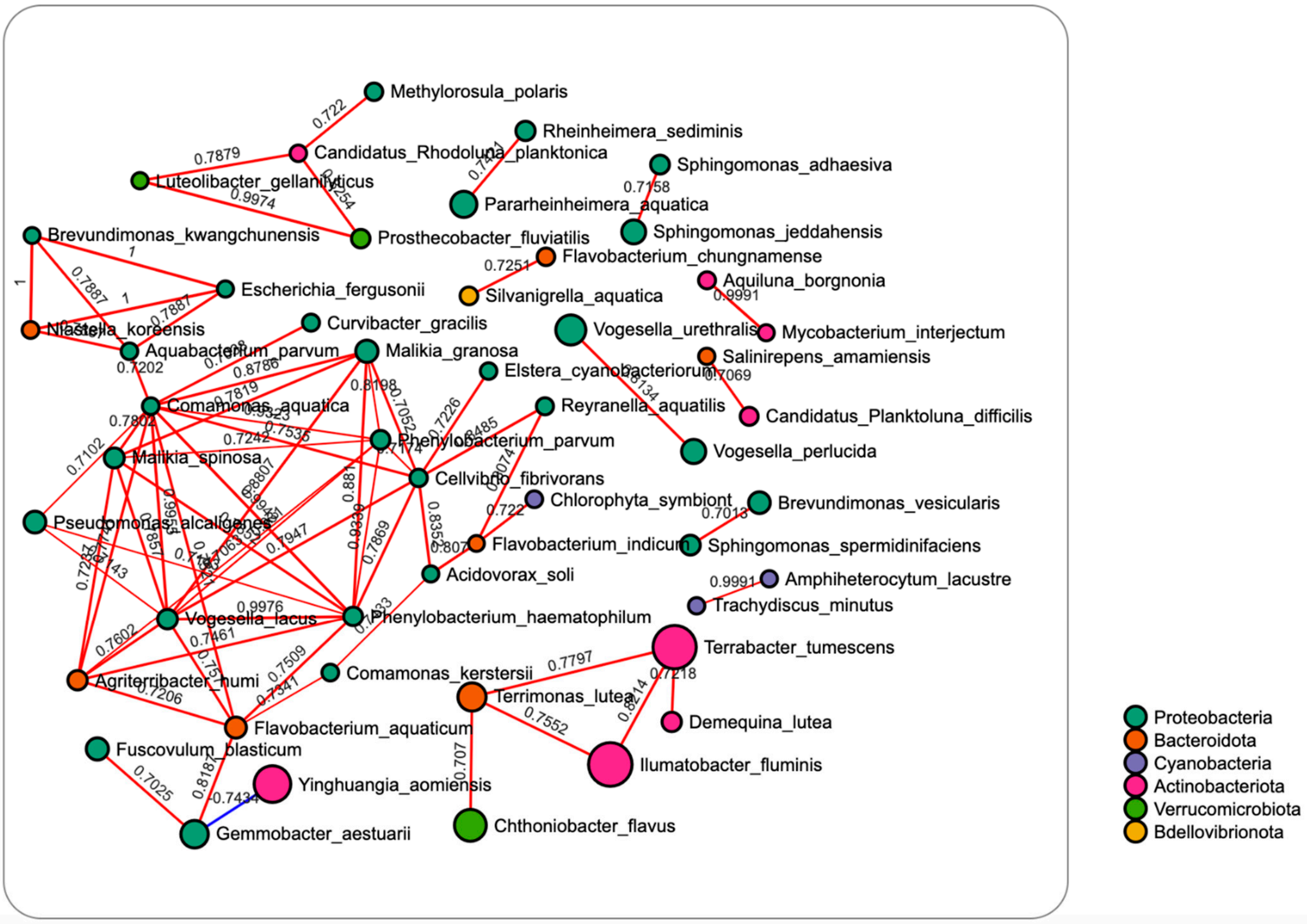

Figure S15. Correlation network based on estimates of taxa co-occurrence at species level in raw surface water samples. The size of each node represents the relative abundance of the corresponding taxa, and the node color indicates the ancestor phylum. Red edges represent a positive correlation, while blue edges represent a negative correlation. The correlation cut-off value was adjusted to ± 0.7

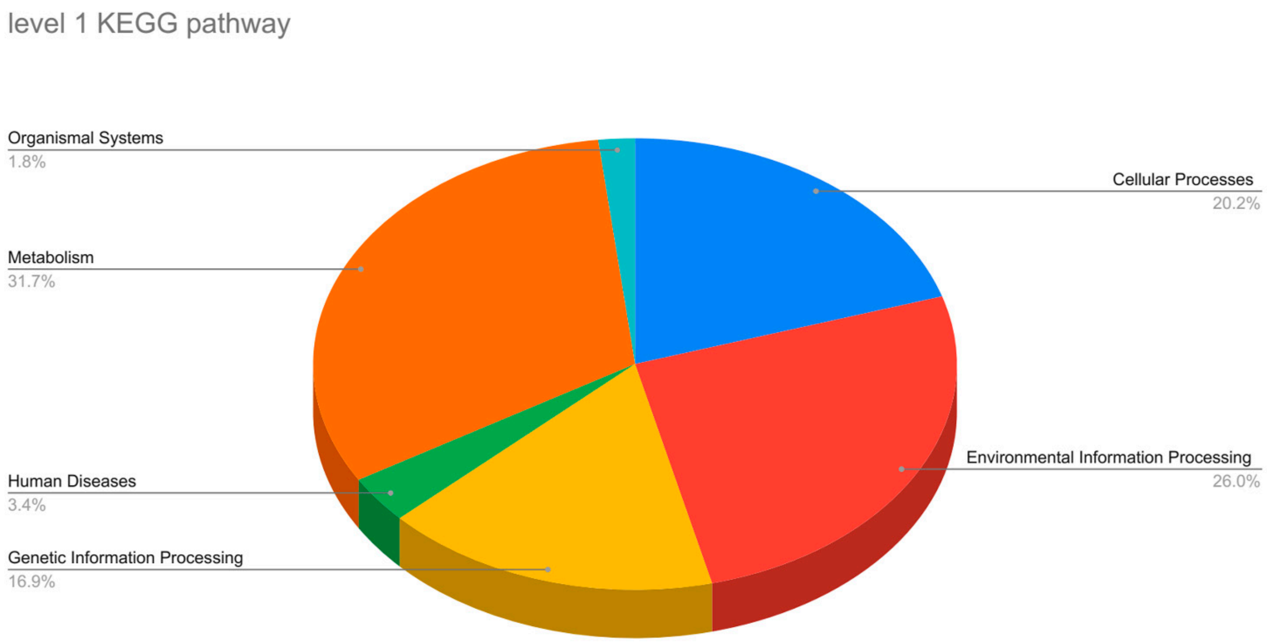

Figure S16. The relative abundance of the primary functional features among bacterial communities in raw surface water samples on level 1 as predicted by Tax4Fun2 tool.

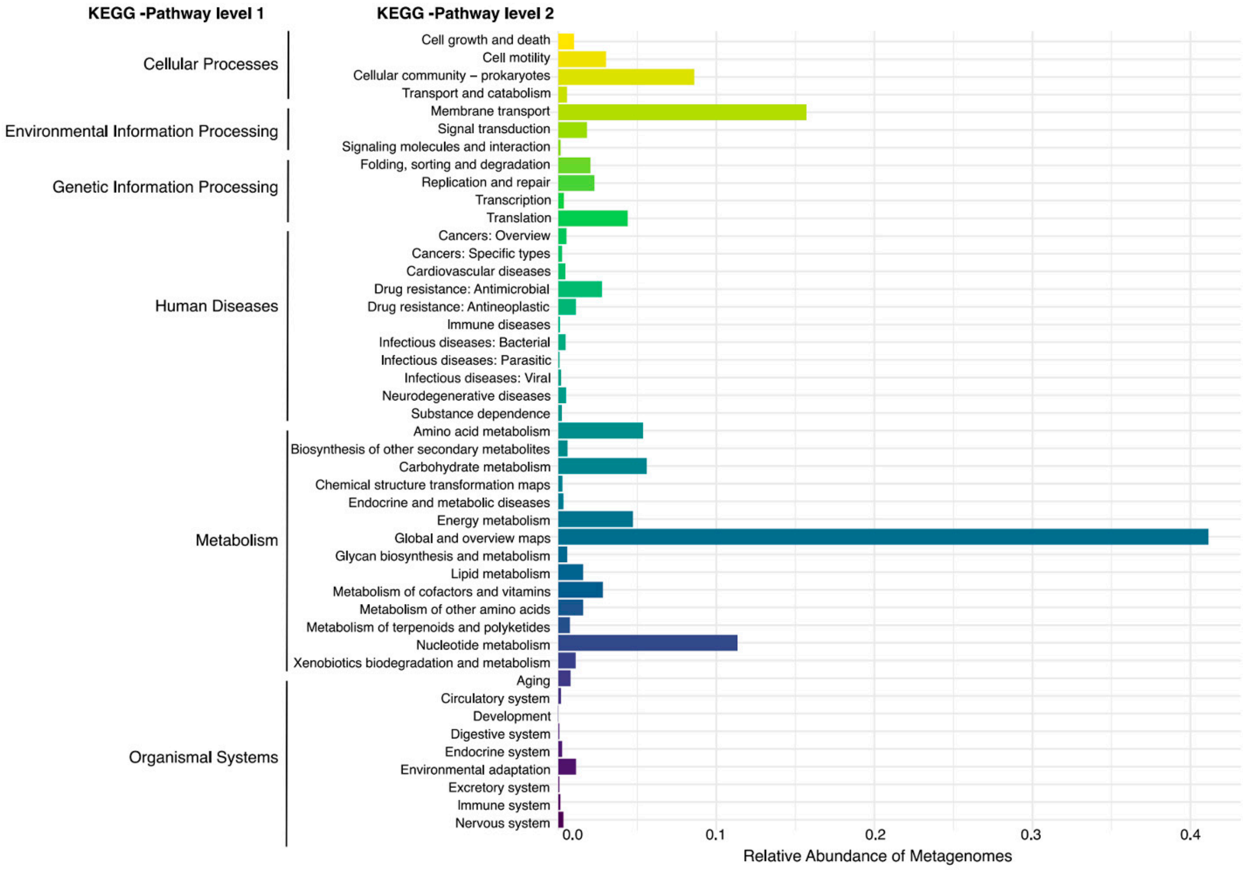

Figure S17. The relative abundance of the primary functional features among bacterial communities in raw surface water samples on level 2 and their corresponding functional features on level 1, as predicted by Tax4Fun2 tool.

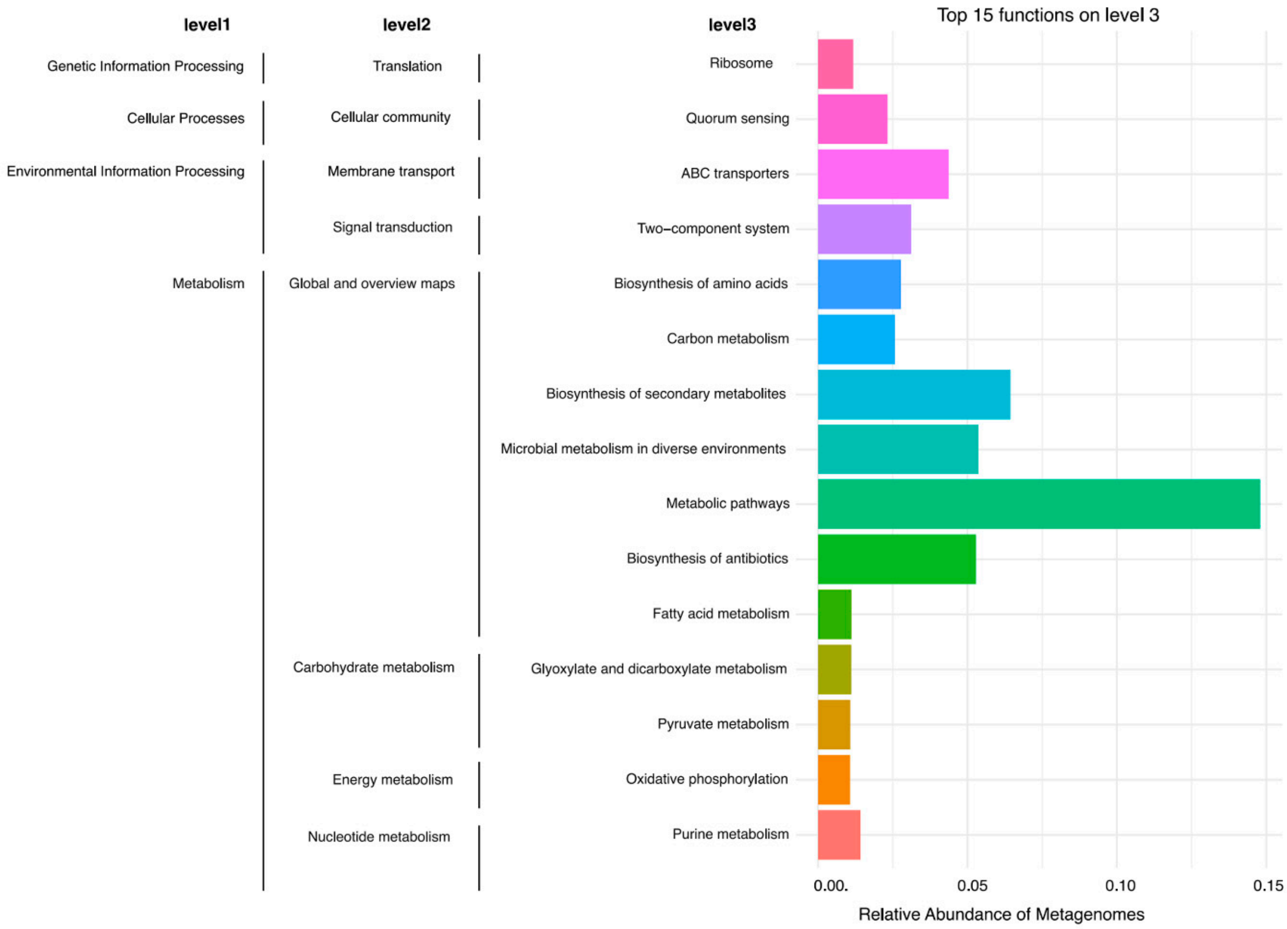

Figure S18. The relative abundance of the top 10 functional features among bacterial communities in raw surface water samples on level 3 and their corresponding functional features on level 1 and 2, as predicted by Tax4Fun2 tool.

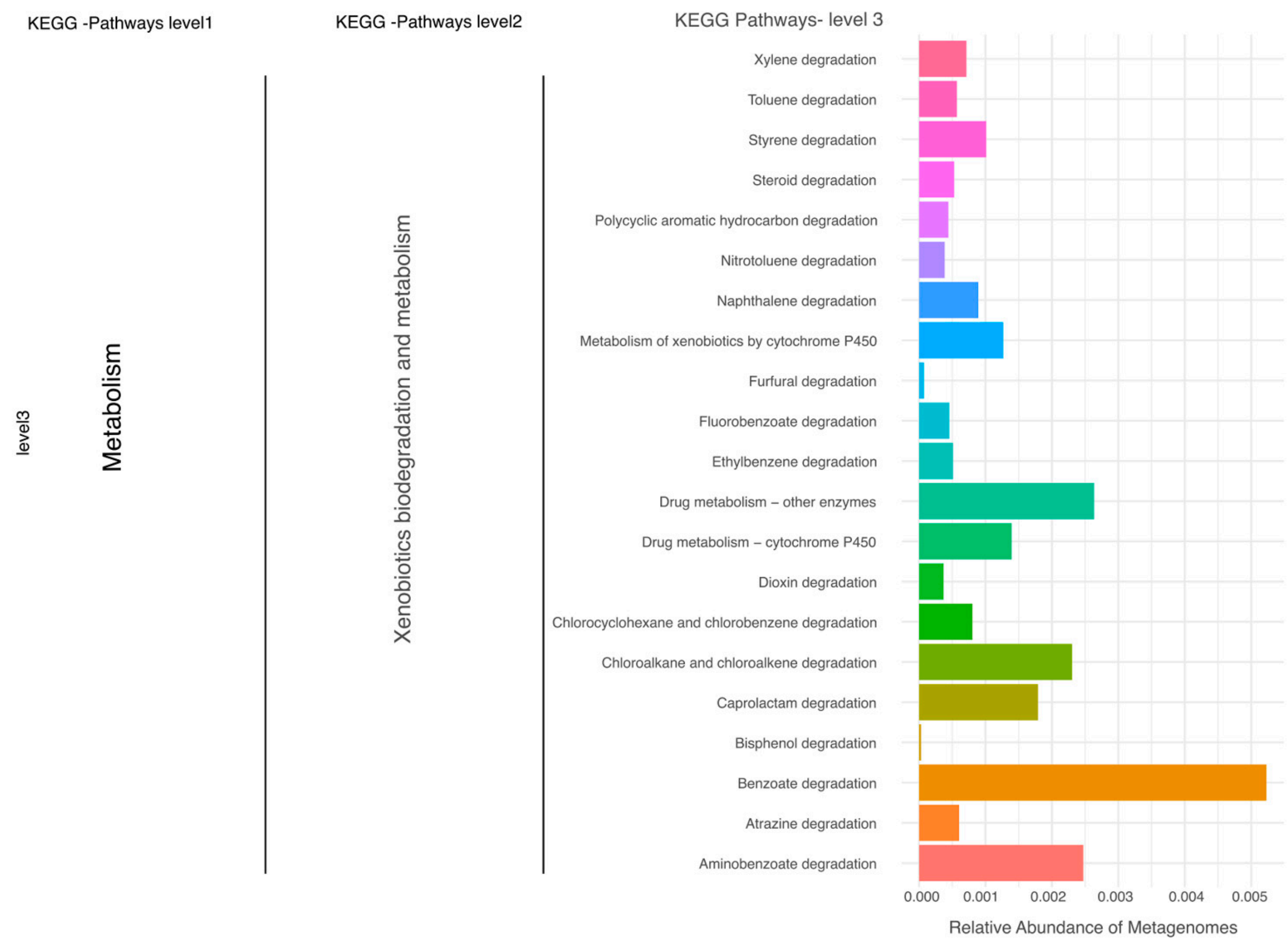

Figure S19. The relative abundance of level 3 KEGG pathways belonging to xenobiotic biodegradation and metabolism and their corresponding functional features on level 1, as predicted by Tax4Fun2 tool.

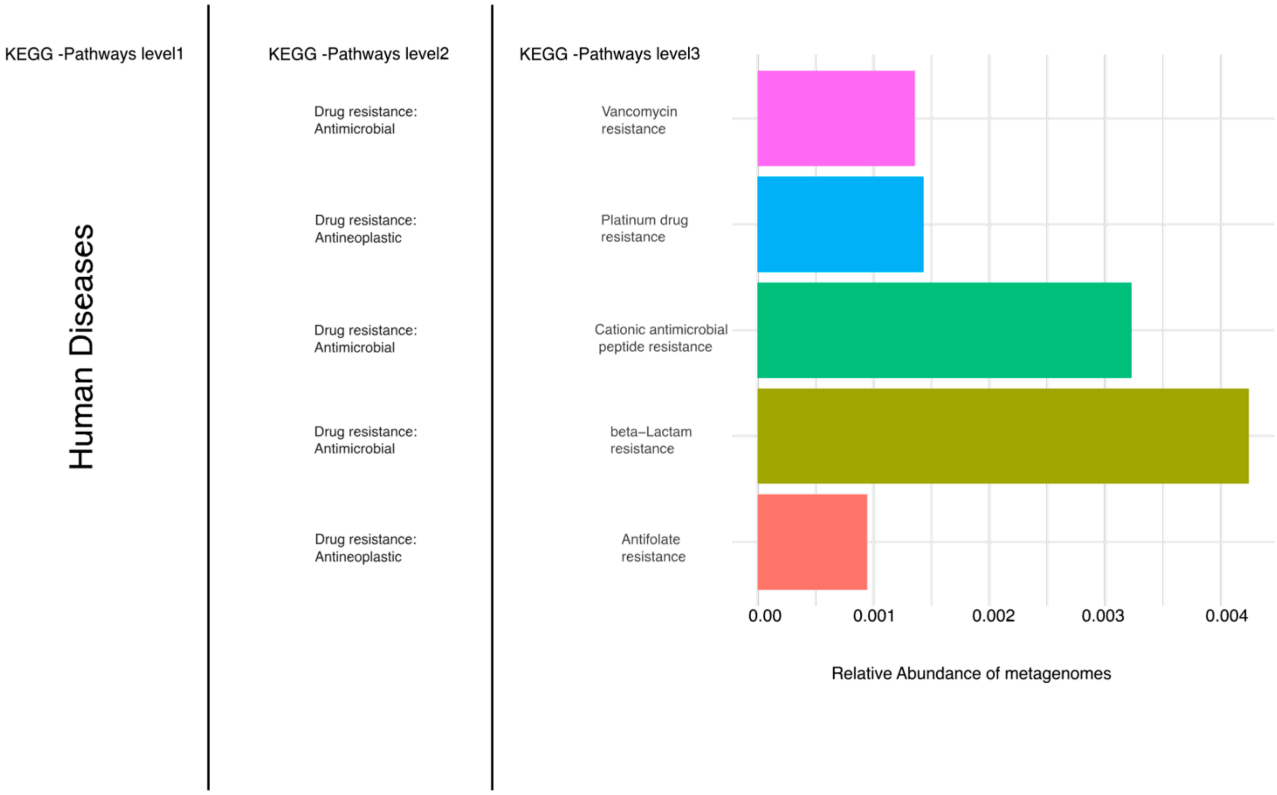

Figure S20. The relative abundance of level 3 KEGG pathways belonging to drug resistance and their corresponding functional features on level 1, as predicted by Tax4Fun2 tool.

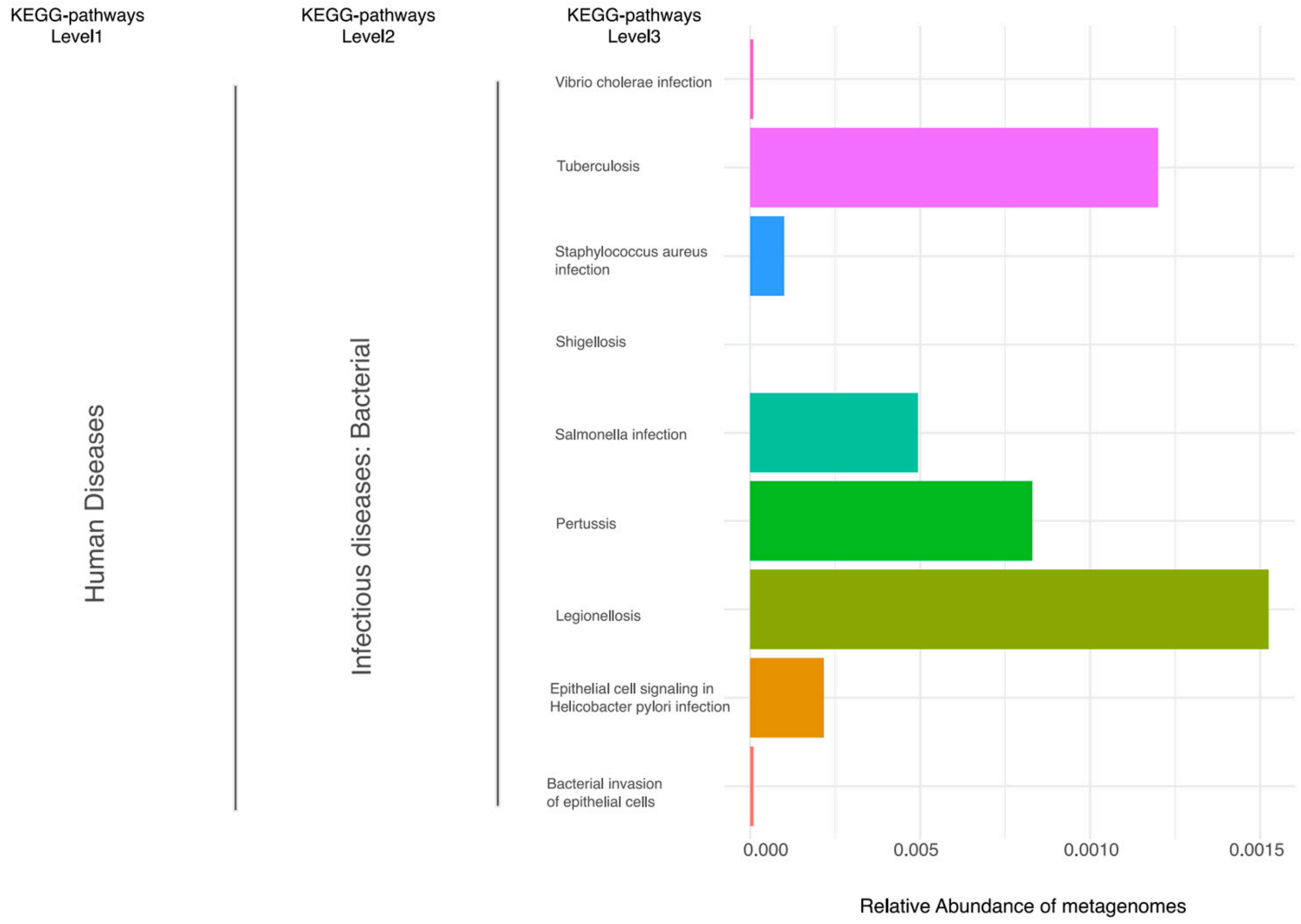

Figure S21. The relative abundance of level 3 KEGG pathways belonging to bacterial infectious diseases and their corresponding functional features on level 1, as predicted by Tax4Fun2 tool.

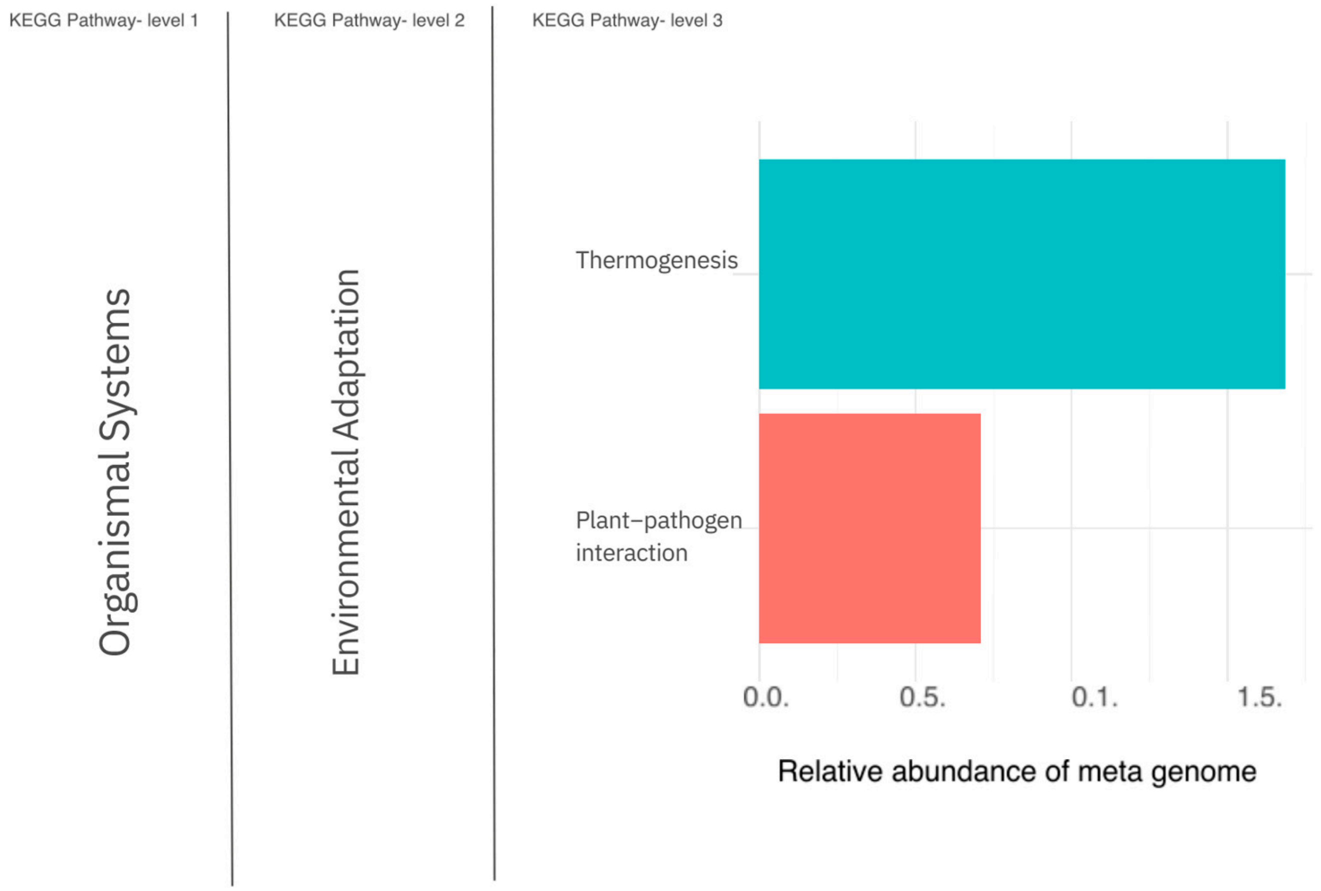

Figure S22. The relative abundance of level 3 KEGG pathways belonging to environmental adaptation and their corresponding functional features on level 1, as predicted by Tax4Fun2 tool.

2. Supplementary Tables

Table S1. Summary of collected raw surface water samples

Sample Number	Water Treatment Station	Date	Description	Source	District	Coordinates
1	Suez Canal Authority	19/5	drinking water outlet	Ismailia canal	Ismailia	30.35° N 32.15° E
2	Eldabaa	19/5	drinking water outlet	Suez canal	Ismailia	30.33° N 32.16° E
3	Elomda Saleh	19/5	drinking water outlet	Suez canal	Ismailia	30.32° N 32.15° E
4	Ein Ghosein	19/5	drinking water outlet	Suez canal	Ismailia	30.30° N 32.18° E
5	Ein Ghosin Elkobra	19/5	drinking water outlet	Sinai canal	Ismailia	30.30° N 32.16° E
6	Elkarnak	19/5	drinking water outlet	Sinai canal	Ismailia	30.30° N 32.15° E
7	Kilo 11	19/5	drinking water outlet	Port-said canal	Ismailia	30.39° N 32.15° E
8	Elfardan	19/5	drinking water outlet	Port-said canal	Ismailia	30.40° N 32.16° E
9	Kilo 7	19/5	drinking water outlet	Port-said canal	Ismailia	30.38° N 32.15° E
10	Mahatet Fayed	18/5	drinking water outlet	Suez canal	Fayed	30.17° N 32.19° E
11	Fanaret Elomda	18/5	drinking water outlet	Suez canal	Fayed	30.22° N 32.17° E
12	Sarabium	18/5	drinking water outlet	Sinai canal	Fayed	30.28° N 32.18° E
13	Sarabium Elraesiya	18/5	drinking water outlet	Suez canal	Fayed	30.25° N 32.18° E
14	Abo Soltan	18/5	drinking water outlet	Suez canal	Fayed	30.24° N 32.18° E
15	Elaqada	18/5	drinking water outlet	Suez canal	Fayed	30.26° N 32.20° E
16	Lisan Alwozaraa	18/5	drinking water outlet	Suez canal	Fayed	30.18° N 32.15° E
17	Adalo	18/5	drinking water outlet	Suez canal	Fayed	30.29° N 32.19° E
18	Abo soir elkobra	6/7	drinking water outlet	Ismailia canal	Abo-soir	30.33° N 32.07° E
19	ElManteqa elsenaeiya	6/7	drinking water outlet	Ismailia canal	Abo-soir	30.34° N 32.12° E
20	Abo dahshan	6/7	drinking water outlet	Ismailia canal	Abo-soir	30.34° N 32.11° E
21	Abo Ayad	6/7	drinking water outlet	Manayef canal	Abo-soir	30.33° N 32.07° E
22	Sabaa Abar Gharbya	6/7	drinking water outlet	Ismailia canal	Abo-soir	30.33° N 32.07° E
23	Manayef	6/7	drinking water outlet	Manayef canal	Abo-soir	30.32° N 32.10° E
24	Qasassin	5/5	drinking water outlet	Ismailia canal	Qasassin	30.33° N 31.56° E
25	Om Azam	5/5	drinking water outlet	Ismailia canal	Qasassin	30.33° N 32.02° E
26	Elmahsama Elqadima	5/5	drinking water outlet	Ismailia canal	Qasassin	30.33° N 31.57° E

Sample Number	Water Treatment Station	Date	Description	Source	District	Coordinates
27	Tal Kebeer	5/5	drinking water outlet	Ismailia canal	Tal Kebeer	30.33° N 31.43° E
28	Wadi Elmolak	5/5	drinking water outlet	Ismailia canal	Tal Kebeer	30.33° N 31.48° E
29	ElBaalwa elsoghra	5/5	drinking water outlet	Ismailia canal	Tal Kebeer	30.32° N 31.53° E
30	Qantara Gharb	23/5	drinking water outlet	Port-said canal	Qantara Gharb	30.50° N 32.18° E
31	Qantara Sharq	23/5	drinking water outlet	Port-said canal	Qantara Gharb	30.51° N 32.18° E
32	Gelbana	23/5	drinking water outlet	Port-said canal	Qantara Gharb	30.50° N 32.19° E
33	Abo Khalifa	23/5	drinking water outlet	Port-said canal	Qantara Gharb	30.44° N 32.15° E
34	Elrayah	23/5	drinking water outlet	Port-said canal	Qantara Gharb	30.50° N 32.18° E
35	Kilo 14	23/5	drinking water outlet	Port-said canal	Qantara Gharb	30.42° N 32.16° E
36	Kilo 19	23/5	drinking water outlet	Port-said canal	Qantara Gharb	30.48° N 32.16° E
37	Shamal Sainaa	23/5	drinking water outlet	Port-said canal	Qantara Gharb	30.51° N 32.18° E
38	—	24/5	Pollution Monitoring Point	AL-Abtal canal	Qantara sharq	30.52° N 32.18° E
39	—	24/5	Pollution Monitoring Point	AL-Abtal canal	Qantara sharq	30.27° N 32.22° E
40	—	24/5	Pollution Monitoring Point	AL-Abtal canal	Qantara sharq	30.27° N 32.24° E
41	—	24/5	Pollution Monitoring Point	AL-Abtal canal	Qantara sharq	30.27° N 32.22° E
42	—	24/5	Pollution Monitoring Point	AL-Abtal canal	Qantara sharq	30.28° N 32.23 E
43	—	24/5	Pollution Monitoring Point	AL-Abtal canal	Qantara sharq	30.29° N 32.23° E
44	—	6/6	Pollution Monitoring Point	Ismailia canal	Tal Kebeer	30.33° N 31.47° E
45	—	6/6	Pollution Monitoring Point	Ismailia canal	Tal Kebeer	30.33° N 31.49° E
46	—	6/6	Pollution Monitoring Point	Ismailia canal	Qasassin	30.33° N 31.56 E
47	—	6/6	Pollution Monitoring Point	Ismailia canal	Qasassin	30.33° N 31.49° E
48	—	6/6	Pollution Monitoring Point	Ismailia canal	Qasassin	30.33° N 32.00° E
49	—	6/6	Pollution Monitoring Point	Ismailia canal	Ismailia	30.34° N 32.15° E
50	—	24/5	Pollution Monitoring Point	Suez canal	Fayed	30.32° N 32.17° E
51	—	24/5	Pollution Monitoring Point	Suez canal	Fayed	30.32° N 32.16° E

Sample Number	Water Treatment Station	Date	Description	Source	District	Coordinates
52	—	24/5	Pollution Monitoring Point	Suez canal	Fayed	30.31° N 32.18° E
53	—	24/5	Pollution Monitoring Point	Suez canal	Fayed	30.28° N 32.19° E
54	—	24/5	Pollution Monitoring Point	Suez canal	Fayed	30.27° N 32.19° E
55	—	24/5	Pollution Monitoring Point	Suez canal	Fayed	30.18° N 32.19° E
56	—	24/5	Pollution Monitoring Point	Suez canal	Fayed	30.16° N 32.21° E
57	—	24/5	Pollution Monitoring Point	Suez canal	Fayed	30.14° N 32.25° E
58	—	24/5	Mahsama sewage station	Station inlet	Fayed	30.27° N 32.21° E
59	—	24/5	Mahsama sewage station	Station outlet	Fayed	30.27° N 32.21° E

Table S2. Physicochemical properties of raw surface water samples.

Sample Number	°C	TDS	PH	BOD	DO	NO3	NO2	NH3	CL	Ca	CaH	Mg	MgH	TH	T.Alk
**Drinking Water Outlets**															
1	26	236	8.3	1.9	6.6	0.07	0.007	0.24	36	26.4	66	9.12	38	104	156
2	26.5	234	7.4	1.8	5.5	N.D	0.009	0.01	34	26.4	66	9.12	38	104	160
3	25.5	234	7.3	3.7	4.6	N.D	0.008	0.11	33	28	70	8.16	34	104	156
4	24.7	238	8	2.7	5.5	N.D	0.006	0.4	34	28	70	9.12	38	108	160
5	25.1	234	7.5	2.8	6.1	0.03	0.008	0.22	35	27.2	68	9.12	38	106	160
6	25	236	7.2	3.3	6.2	N.D	0.007	N.D	35	28	70	10.1	42	112	158
7	26.7	238	7.5	1.7	5.5	N.D	0.007	0.35	35	26.4	66	11	46	112	164
8	27	238	7.4	3.9	5.1	N.D	0.007	N.D	35	27.2	68	10.1	42	110	162
9	26.8	234	7.1	1.7	5.3	N.D	0.006	0.2	34	28	70	10.3	43	113	164
10	24.8	236	7.1	0.7	5.6	N.D	0.001	0.02	30	20.8	52	13	54	106	148
11	24.9	234	7	2.8	5.7	N.D	0.002	0.06	35	22.4	56	10.6	44	100	134
12	24.8	234	7.4	2.4	5.6	0.02	0.001	0.08	36	23.2	58	11.5	48	106	154
13	25.1	236	7.2	3.7	5.2	N.D	0.003	0.1	38	24	60	10.6	44	104	166
14	25.5	236	7.3	2.2	5.2	N.D	0.002	0.01	34	19.2	48	14.4	60	108	162
15	25.4	234	7.5	4.4	4.6	0.03	0.001	0.03	33	22.4	56	11.5	48	104	160
16	24.9	238	7.3	4.6	6.4	0.03	0.001	0.2	36	23.2	58	11.5	48	106	162
17	25.2	238	7.2	3.23	5.2	0.37	0.003	0.02	35	24	60	10.1	42	102	160
18	28	234	7.7	4.3	6	0.24	0.012	0.34	43	24	60	14.4	60	120	180
19	28.2	236	7.4	3.6	7	0.31	0.011	0.35	40	24	60	13.4	56	116	178
20	27	228	7.2	2.6	6.1	0.35	0.012	0.36	45	26.4	66	11	46	112	172
21	26.5	236	7.2	2.2	4	0.18	0.013	0.32	43	24	60	11	46	106	182
22	25.6	238	7.2	3.4	5.2	0.26	0.01	0.3	45	25.6	64	8.64	36	100	180
23	26.1	236	7.6	0.4	5	1.3	0.01	0.3	43	25	60	8.5	46	106	176
24	24.5	234	7.7	2.6	6.2	0.59	0.01	0.15	37	22.4	56	14.9	62	118	124
25	24.6	236	7.6	2.8	5.4	0.67	0.008	0.21	38	23.2	58	14.9	62	120	156
26	24.3	238	7.8	4	4.1	0.57	0.009	0.22	39	23.2	58	12.5	52	110	168
27	25.1	212	7.8	2.5	3.5	0.67	0.001	0.13	39	22.4	56	13.4	56	112	154
28	24.8	210	7.9	1.6	6	0.63	0.002	0.06	40	21.6	54	14.4	60	114	160
29	24.9	212	8	1.3	5	0.55	0.004	0.11	36	21.6	54	14.4	60	114	158
30	25	234	8.4	2	6.9	0.57	0.008	0.25	32	24	60	12.5	52	112	150
31	25.4	236	8.3	1.2	6.7	0.49	0.009	0.15	33	24	60	12	50	110	160
32	25.3	238	8.1	1.7	6.9	0.52	0.014	0.36	34	24	60	11.5	48	108	154
33	25.3	234	8.2	1.9	5.9	1.05	0.006	0.13	31	24.8	62	11.5	48	110	150
34	25.8	236	8.4	2.8	6.7	1.3	0.01	0.26	32	24	60	13	54	114	154
35	25.1	236	8.1	2.2	4.5	1.5	0.01	0.2	31	24	60	12	52	112	154
36	25.6	234	8.5	2.9	4.5	0.46	0.011	0.2	29	23.2	58	12	50	108	152
37	25.6	234	8.4	2.7	4.9	0.23	0.012	0.19	32.5	24	60	13	54	114	156
**Pollution Monitoring Points**															
38	26	236	6.7	1.7	6.6	0.34	0.005	0.13	32.5	24	60	13	54	114	156

Sample Number	°C	TDS	PH	BOD	DO	NO3	NO2	NH3	CL	Ca	CaH	Mg	MgH	TH	T.Alk
39	25.9	234	8.4	1.2	8	0.59	0.331	0.32	50	25.6	64	13.4	56	120	88
40	25.4	234	8	3.5	6.3	0.43	0.336	1.08	53	28.8	72	14	160	232	188
41	25.8	236	7.5	2.9	6.15	N.D	0.345	0.32	51	28	70	14.3	162	232	180
42	25.8	234	7.7	1.1	8	0.35	0.345	0.85	54	28	70	13.8	160	230	176
43	25.7	234	7.8	2.1	6.4	0.43	0.354	0.69	50	22.4	56	12.7	178	234	182
44	26.1	227	8.2	2.6	6.7	0.39	0.01	0.21	29	22.4	56	13	54	110	150
45	26.3	218	8.4	2.9	6.6	0.5	0.01	0.22	26	22.4	56	13.4	56	112	164
46	26.3	233	8.6	3.8	5.9	0.34	0.012	0.36	30	24	60	11.5	48	108	166
47	26.8	230	8.2	4	5.1	0.61	0.01	0.37	26	26.4	66	10.1	42	108	160
48	25.9	227	8.5	3.9	6.5	0.29	0.009	0.25	26	24.8	62	10.6	44	106	180
49	26.1	234	8	2.2	2.4	0.14	0.009	0.28	29	22.4	58	13	56	114	150
50	25.9	232	7.2	2.7	6	0.35	0.012	0.24	29	25.6	64	13.4	56	120	160
51	25.8	232	7.4	2	6.4	0.5	0.005	0.18	30	26.4	66	10.1	42	108	160
52	25.7	232	7.2	3.5	5.2	0.2	0.007	1.18	29	25.6	64	12	50	114	158
53	25.9	232	7.6	2	6	0.52	0.011	0.22	30	24.8	62	13	54	116	162
54	25.9	232	7.2	3.1	6.3	0.11	0.011	0.2	30	24	60	13.4	56	116	164
55	25.4	232	7.4	2.5	6.4	0.37	0.1	0.3	30	25.6	64	12.5	52	116	158
56	25.9	232	7.2	3.6	6.2	0.63	0.008	0.42	28	24	60	12.5	52	112	160
57	26.1	231	7.6	1.5	6.1	0.7	0.007	0.23	28	24.8	62	12.5	52	114	158
**Sewage treatment station points**															
58	26.2	**	**	4.4	2.3	**	**	**	**	**	**	**	**	**	**
59	26.4	**	**	5.9	0.6	**	**	**	**	**	**	**	**	**	**

TDS, Total dissolved solids; BOD, Biochemical oxygen demand; DO, Dissolved oxygen; NH_3, ammonia; NO_3, nitrate; NO_2, nitrite; Cl, chloride; CaH, Calcium hardness; MgH, Magnesium hardness; Ca, Calcium; Mg, Magnesium; TH, Total hardness; T.Alk, Total alkalinity; ** Not analyzed as per Egyptian Standard guidelines; N.D. not available.
